# In Vitro Interactions between Okadaic Acid and Rat Gut Microbiome

**DOI:** 10.3390/md20090556

**Published:** 2022-08-30

**Authors:** Yang Liu, Siyuan Xu, Qiudie Cai, Dawei Li, Hongye Li, Weidong Yang

**Affiliations:** Key Laboratory of Aquatic Eutrophication and Control of Harmful Algal Blooms of Guangdong Higher Education Institute, College of Life Science and Technology, Jinan University, Guangzhou 510632, China

**Keywords:** okadaic acid, microbiota, fermentation, metabolism

## Abstract

Okadaic acid (OA) is a marine biotoxin associated with diarrhetic shellfish poisoning (DSP), posing some threat to human beings. The oral toxicity of OA is complex, and the mechanism of toxicity is not clear. The interaction between OA and gut microbiota may provide a reasonable explanation for the complex toxicity of OA. Due to the complex environment in vivo, an in vitro study may be better for the interactions between OA and gut microbiome. Here, we conducted an in vitro fermentation experiment of gut bacteria in the presence of 0–1000 nM OA. The remolding ability of OA on bacterial composition was investigated by 16S rDNA sequencing, and differential metabolites in fermentation system with different concentration of OA was detected by LC-MS/MS. We found that OA inhibited some specific bacterial genera but promoted others. In addition, eight possible metabolites of OA, including dinophysistoxin-2 (DTX-2), were detected in the fermentation system. The abundance of *Faecalitalea* was strongly correlated with the possible metabolites of OA, suggesting that *Faecalitalea* may be involved in the metabolism of OA in vitro. Our findings confirmed the direct interaction between OA and gut bacteria, which helps to reveal the metabolic process of OA and provide valuable evidence for elucidating the complex toxicity of OA.

## 1. Introduction

Diarrhetic shellfish poisoning (DSP) toxin is a class of liposoluble marine phycotoxin, distributed around the world, that can be highly concentrated in shellfish and cause severe gastrointestinal symptoms in human beings through the food chain [1,2]. According to statistics, over 25,000 people have been poisoned by DSP in the past 50 years, although no deaths have been reported [3]. Symptoms of poisoning caused by DSP toxins are similar to gastrointestinal diseases, and acute symptoms usually disappear within 2–3 days. As a result, human DSP events are easily ignored, and their actual incidence may be much higher than reported [4]. The association between consumption of shellfish contaminated with DSP toxins and increased incidence of colorectal cancer makes DSP toxins one of the most concerning toxins [5,6].

**Figure 1:**
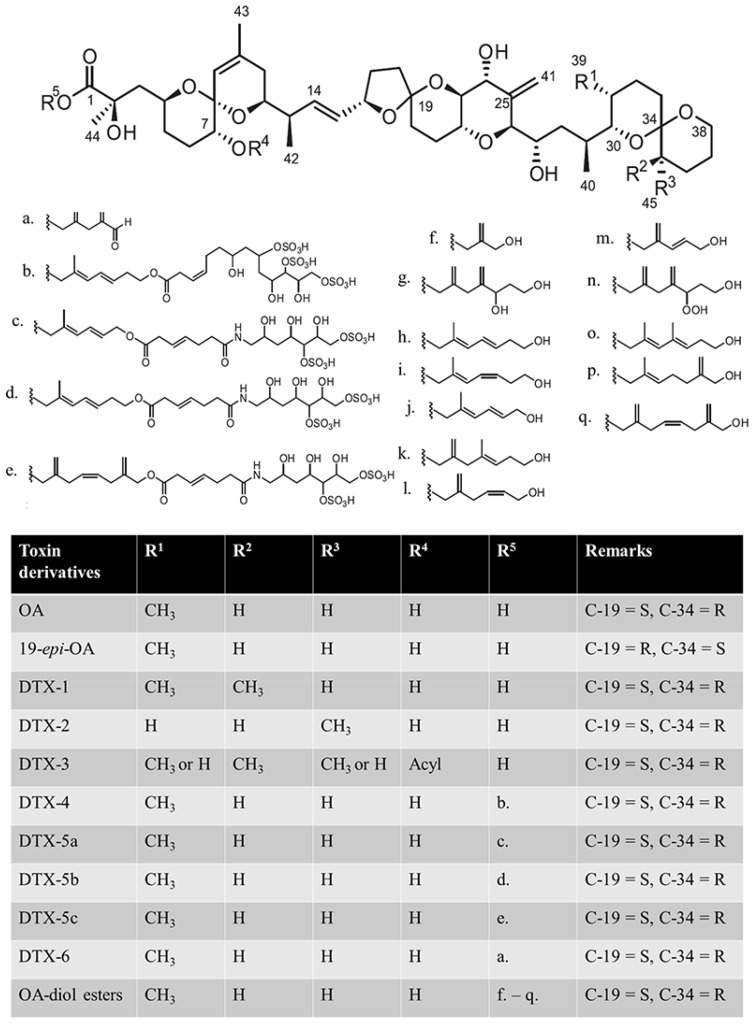
Structures of okadaic acid (OA), dinophysistoxins (DTXs), and their derivatives. C-19 and C-34 denote the 19th carbon and 34th carbon atoms, respectively; S and R denote the anticlockwise and clockwise stereochemistry of the carbon, respectively [7]. (**a**) C_7_H_9_O; (**b**) C_22_H_37_S_3_O_17_; (**c**) C_20_H_34_S_2_O_4_N; (**d**) C_22_H_36_S_2_O_14_N; (**e**) C_24_H_36_S_2_O_14_N; (**f**) C_4_H_7_O; (**g**) C_9_H_15_O_2_; (**h**,**i**) isomer of C_8_H_13_O; (**j**) C_7_H_14_O; (**k**) C_9_H_15_O; (**l**) C_7_H_11_O; (**m**) C_6_H_9_O; (**n**) C_9_H_15_O_3_; (**o**,**p**) isomer of C_9_H_15_O; (**q**) C_10_H_15_O.

DSP toxins include okadaic acid (OA), dinophysistoxins (DTXs), and other derivatives, as reviewed by Lee et al. (Figure 1) [7]. The main toxins causing DSP poisoning include OA, DTX-1, DTX-2 and DTX-3, among which DTX-3 is a group of acylated derivatives of DTX-1 that cause intoxication by transforming back to DTX-1 in the gastrointestinal tract of consumers [8,9,10]. Some intraperitoneal (i.p.) administration studies have shown that OA has similar toxicity to DTX1, while DTX2 is less potent [11,12,13]. Based on the i.p. toxicity, the European Food Safety Authority (EFSA) established toxicity equivalency factors (TEFs): OA = 1, DTX-1 = 1, DTX-2 = 0.6 [8]. However, oral administration studies have reached a different conclusion: DTX-1 > OA > DTX-2 [14,15]. There may be many reasons for this discrepancy, such as differences in intake efficiency, metabolic conversion of toxins, etc., which remains to be verified.

OA is widely used in toxicological studies concerning DSP toxins both in cell lines and in vivo [7,16]. As a potent inhibitor of serine/threonine protein phosphatases (PPs), the inhibition of PP1 and PP2A is considered to be the main toxic mechanism of OA [17,18,19,20]. However, this fails to explain all the toxic effects of OA, especially the large fluctuations in oral toxicities in vivo [21,22]. The metabolism of OA in different cell lines has been reported successively [23,24]. Some possible metabolites of OA, such as DTX-2, have also been detected in the feces of rats exposed to OA [25]. It is likely that the differences in the ability of different cell lines and individuals to metabolize OA are important reasons for the differences in DSP toxicity.

The intestinal tract is the preliminary metabolic place of xenobiotics in the host, and the bacteria in it play an important role in the metabolism of xenobiotics, which to some extent provides an explanation for inter-individual differences in drug efficacy [26]. More importantly, many xenobiotics or their metabolites can significantly alter the composition and function of gut bacteria, and thus change the toxicity of drugs or xenobiotics [27,28]. Previous studies have shown that OA exposure leads to swollen intestine and damaged epithelium [29,30], which may provide more opportunities for opportunistic pathogens in gut lumen. Increased abundance of *E. coli* in gut lumen has been proved to result in a significant increase in the toxicity of OA [31]. The potential bacterial metabolism of OA is likely to be a non-negligible factor affecting the toxicity of OA. Studies on interaction between OA and gut bacteria can help explain the complexity and fluctuations in OA toxicity. Our previous study demonstrated that OA exerted a great effect on gut bacteria, featuring enrichment of specific bacterial genera and significant changes in bacterial metabolism genes [25]. However, the possibility of OA metabolites entering feces through enterohepatic circulation after biotransformation in the liver are un-excludable, and the direct evidence of the interaction between OA and gut bacteria is still lacking.

Though about 80% of the bacteria in the human gut are unknown and unculturable, in vitro fermentation of the gut bacteria can still help understand the relationship between gut microbiota and human health [32,33]. A recent study showed that some species of lactic acid bacteria can reduce OA content and toxicity in vitro [34], suggesting the direct potential metabolic ability of OA by gut bacteria. Gifu anaerobic medium (GAM) is a standardized medium for gut bacteria in vitro [35]. To further clarify whether OA is metabolized in the intestine and to understand the bacteria involved in OA metabolism, in vitro fermentation of gut bacteria with different concentrations of OA was carried out using GAM. The changes in bacterial composition and metabolites in the fermented system were analyzed by 16S rRNA high-throughput technology and metabolomics. Our study may provide new insights into the complex toxicity of OA.

## 2. Results

### 2.1. Effects of OA on Bacterial Diversity In Vitro

After separation by the modified sterilized normal saline solution, the fecal microbes were cultured in the GAM fermentation medium with final concentrations of 0, 50, 100, 250 and 1000 nM OA in an anaerobic environment. The effects of OA on bacterial alpha and beta diversity were analyzed at 24 h using 16S rRNA high-throughput technology. A total of 1,881,621 paired-end raw reads from bacterial 16S regions were obtained from 15 fermented samples. After filtration, combination and quality control, an average of 98,967 valid tags were obtained and assigned to 743 OTUs following 97% similarity cutoff assignment. An average of 259 ± 21 OTUs were obtained in each sample.

**Figure 2:**
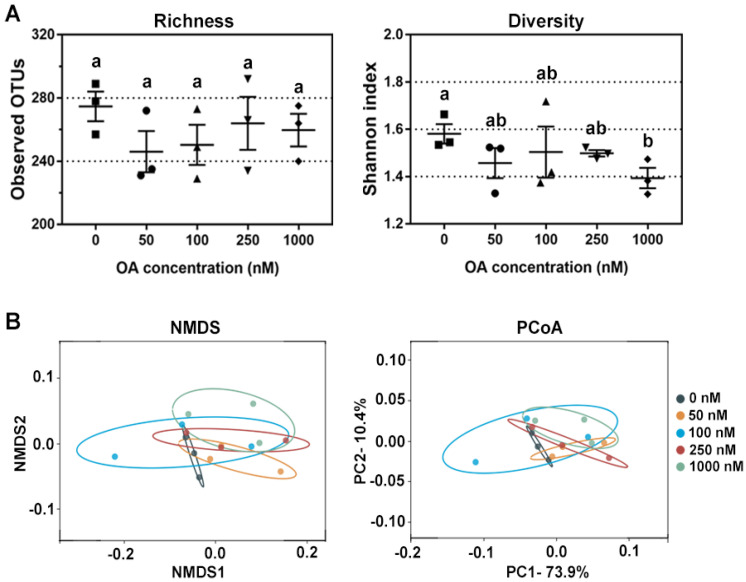
Changes in bacterial diversity after OA exposure in vitro. (**A**) Comparison of bacterial richness and diversity after exposure to 50 nM, 100 nM, 250 nM and 1000 nM of OA for 24 h compared to the control group (*n* = 3). All data represent the mean ± SEM. Dot plots of square, circle, triangle, inverted triangle and rhombus indicate data from individual sample of control, 50 nM, 100 nM, 250 nM and 100 nM group, respectively. a, b, and ab are statistical results of difference among groups, and groups with same letter represents no significant difference. (**B**) Bacterial β-diversity revealed by NMDS and PCoA after 0 nM, 50 nM, 100 nM, 250 nM and 1000 nM OA exposure in vitro.

Then, the alpha-diversity (richness and diversity) of the bacterial community was evaluated by the observed OTUs and Shannon indexes, respectively. The richness of the bacterial community was not affected, but the diversity of the bacterial community decreased in a dose-dependent manner (Figure 2A). Based on the weighted unifrac distance matrix, principal coordinate analysis (PCoA) and nonmetric multidimensional scaling (NMDS) analysis were used to evaluate β-diversity of bacterial community among groups. As shown in Figure 2B, differences in bacterial composition were larger in the treatments with higher concentrations of OA. The analysis of variance using distance matrices (Adonis) and analysis of molecular variance (Amova) were used to quantify the difference between the OA-exposed groups and control group (0 nM), and the results were similar but without statistical significance (Table 1).

### 2.2. Changes in Bacterial Genera after OA Exposure

**Figure 3:**
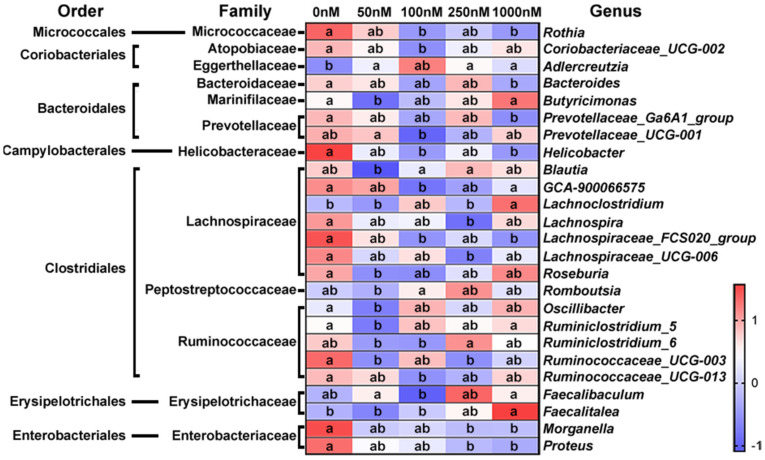
Significantly changed genera after exposure to OA at different concentrations. The relative abundance of bacteria at each time point was visualized as the mean value within the corresponding group after homogenization by Z-score (*n* = 3). a, b, and ab are statistical results of the differences among groups, and groups with the same letter represent not significant differences.

The most abundant sequence in each OTU was selected as the representative sequence to annotate taxonomic information through RDP. The identified OTUs with clear taxonomic information belong to 12 phyla, 17 classes, 22 orders, 38 families, 90 genera and 16 species. The relative abundance of some bacteria fluctuated at different taxonomic levels after OA exposure. Most of the changing bacteria were concentrated in a few taxa, so we focused on the genera that could provide more valuable information. As shown in Figure 3, the relative abundance of 25 genera was significantly changed after OA exposure, and most of them belong to Clostridiales. Overall, the changes in bacterial abundance caused by OA exposure were very complex, and some of them showed a dose-dependent relationship. The abundance of *Rothia*, *Bacteroides*, *Prevotellaceae* Ga6A1 group, *Helicobacter*, GCA900066575, *Lacnchnospiraceae* FCS020 group, *Morganella* and *Proteus* was decreased significantly, while that of *Lanchnoclostridium* and *Faecalitalea* was increased.

### 2.3. Metabolites of OA in the Fermentation System

**Figure 4:**
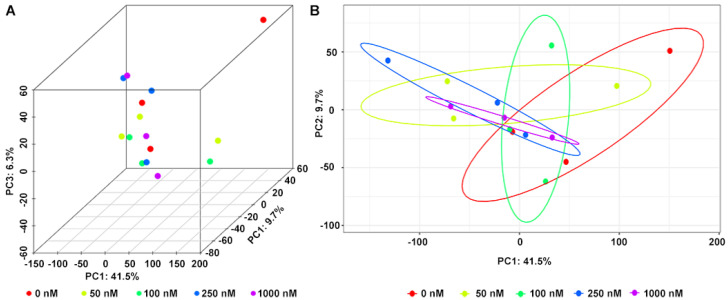
Principal component analysis (PCA) of metabolites in fermentation system at different concentrations of OA in three (**A**) and two (**B**) dimensions.

In addition to 16S rRNA analysis of bacterial community, the OA metabolites in the fermentation system were analyzed by LC-MS/MS. In total, 13,707 peaks were obtained from mass spectrometer (6512 in negative and 7195 positive ion modes). After filtering noise according to relative standard deviation, the data of 12,002 peaks were normalized for principal component analysis (PCA). As shown in Figure 4, the metabolites were visualized in both three and two dimensions, and there were small changes in metabolite composition under different OA concentrations.

**Figure 5:**
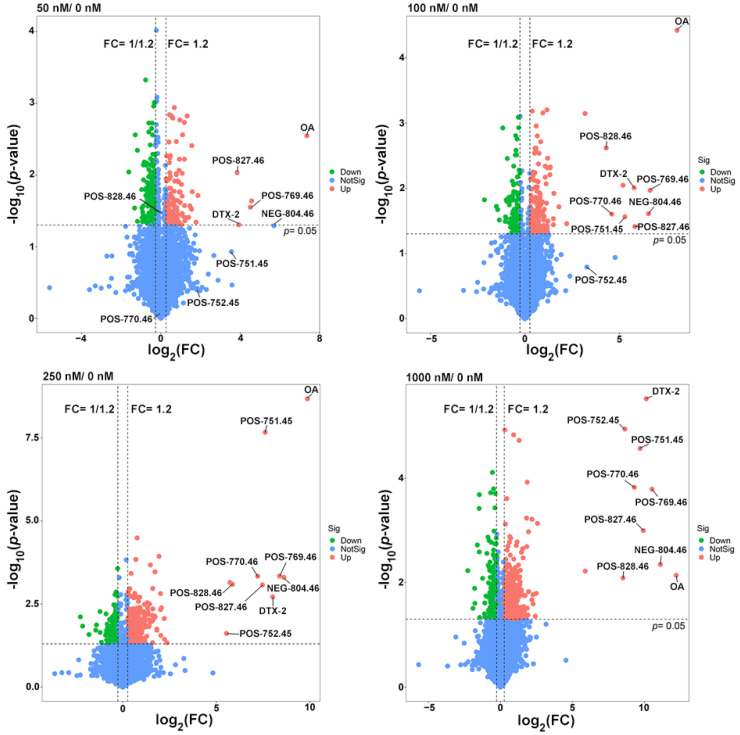
Volcano plots of differential metabolites in the 50, 100, 250 and 1000 nM OA groups compared to the control group. The missing value was temporarily filled with half of the minimum value, and the normalized peak area of each metabolite by peak area of the interior standard was used to visualize in the volcano map.

Therefore, more attention was paid to the study of differential metabolites among the OA-exposed groups. A total of 1181 differential metabolites (from the OA exposure groups and control together) were found after a fold-change (FC) ≥ 1.2 and *p* ˂ 0.05 set up for statistically significant differences (Figure 5), among which 31 were the shared differential metabolites. Although most of them did not match explicit annotated information in the secondary MS, they may be involved in the metabolic process of OA (Table 2).

Therefore, we paid more attention to the study of common differential metabolites between OA-exposed groups. When fold-change (FC) ≥ 1.2 and *p* ˂ 0.05 were set for significant difference, there were 1181 differential metabolites in the combined count between the OA exposure groups and the control group (Figure 5), among which 31 were the shared differential metabolites. Although most of them did not match the explicit annotated information in the secondary MS, they may be involved in the metabolic process of OA (Table 2).

### 2.4. Potential OA Metabolites and Correlated Bacteria

**Figure 6:**
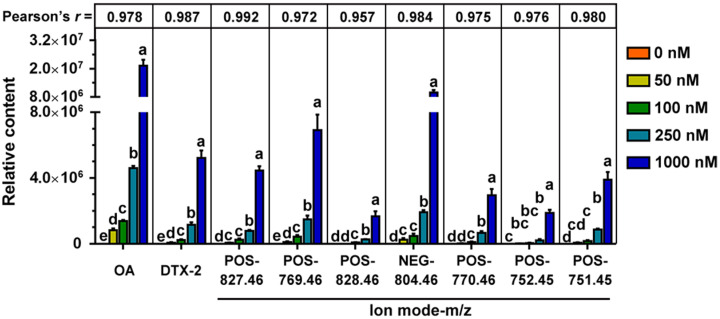
Relative content of the potential metabolites of OA not found in the control group (*n* = 3), and their correlation with OA concentration revealed by Pearson test. Letters are statistical results of difference among groups, and groups with same letter represents no significant difference.

Given that metabolites not present in the control group (0 nM group) but closely associated with OA concentration may be important participants in OA metabolism, we further marked them in the volcano map, including DTX-2, POS-827.46, POS-769.46, POS-828.46, NEG-804.46, POS-770.46, POS-752.45 and POS-751.45 (Figure 5). A total of nine metabolites were absent in the control group, among which four (including OA) were in the 31 shared differential metabolites. The contents of these nine compounds were highly correlated with the exposure concentrations of OA, and the correlations were all over 0.95 according to the Pearson test (Figure 6). Other shared differential metabolites showed some correlation with OA concentration to varying degrees, which may be involved in OA metabolism (Supplementary Figure S1).

**Figure 7:**
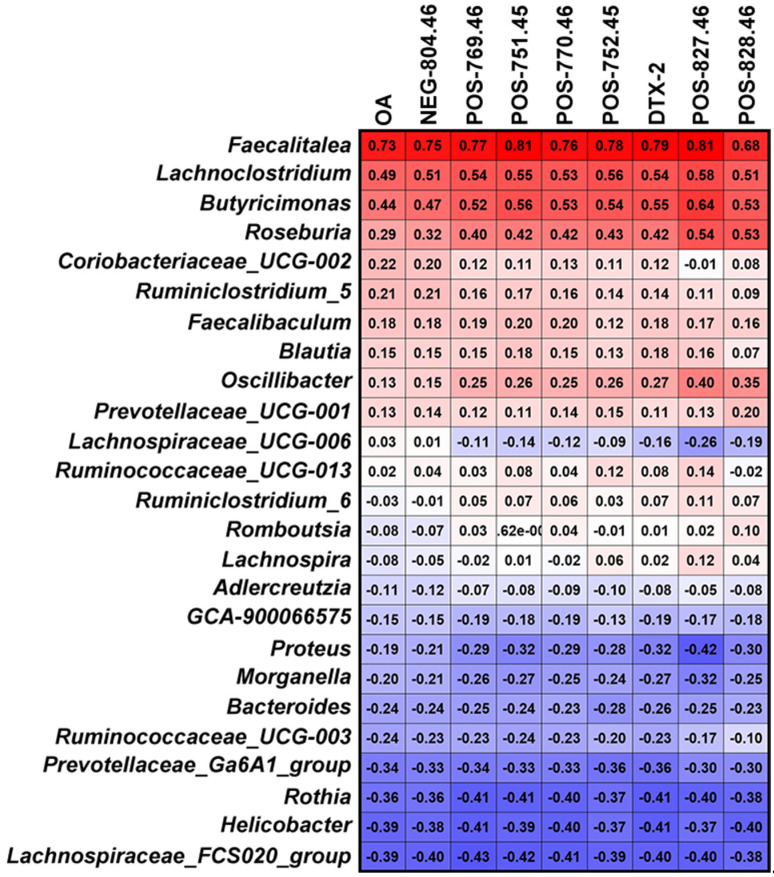
Spearman correlation test between the significantly affected bacterial genera and OA metabolites.

To further explore information for bacteria involved in OA metabolism, a Spearman correlation analysis was performed between abundance of the 25 significant affected bacterial genera and the main potential metabolites of OA (Figure 7). The results showed that the genera *Faecalitalea*, *Lachnoclostridium*, *Butyricimonas* and *Roseburia* had some correlations with OA metabolism.

## 3. Discussion

OA is a liposoluble marine phycotoxin with good thermal and freezing stabilities. Conventional cooking or freezing for 1 month showed limited detoxification effect on OA, making humans vulnerable to OA, leading to DSP, through the food chain [36,37]. The ingested OA was quickly distributed throughout the body and accumulated in specific organs, such as intestinal tissue and stomach [29,38]. OA could still be detected in feces four weeks after oral administration [29]. Therefore, the acute and chronic toxicities of OA have been a major concern in recent decades [16,39]. The gut microbiota plays an important role in the metabolic detoxification of exogenous compounds through direct chemical modification or bioactivation [26], so it may participate in the metabolism of OA by the same token. However, few studies have focused on the interaction of OA and gut microbiota, and even the metabolism of OA in mammals has rarely been mentioned.

In our previous studies, we observed the interaction between OA and gut microbiota, and detected some possible metabolites of OA in the feces of orally OA-exposed rats [25,40]. These results suggest that the gut microbiota is involved in the metabolism of OA, which may be an important factor in the complexity and difference among individuals, although direct evidence is lacking. To verify the direct interaction of OA and gut microbiota, we performed fecal microbial fermentation with OA in vitro. The concentration of OA required to inhibit cell activity was as low as 50 nM, but varied among cell lines [18,41,42,43]. To the best of our knowledge, whether OA has a direct effect on bacteria has not yet been reported. Given that bacteria are thought to be more tolerant than cell lines due to the cell wall, the concentrations of OA were set at 50 to 1000 nM. GAM is a standard medium for the culture of major species in the gut microbiota. Compared with other commonly used media, GAM has simple preparation steps and is suitable for the comparison of bacterial metabolism [35]. To maximize the possible bacterial diversity and compare the bacterial metabolism, the GAM was selected in the 24 h fermentation experiment of this study.

Due to the limitations of fermentation techniques, most bacterial species cannot be cultured in vitro, so the observed OTUs were much lower than those observed in vivo. However, OA at different concentrations tended to reduce the bacterial diversity, especially in the 1000 nM group (Figure 2A). The obtained results indicated that OA had a shaping ability on bacterial community, and also suggested that OA had direct toxicity on specific bacteria. However, different bacteria displayed different toxicity sensitivity to OA. NMDS and PCoA analysis showed that OA did not have a broad-spectrum inhibition on bacterial composition in fermentation system (Figure 2B). OA not only showed an inhibitory effect on certain genera, it also showed some promotional effect on the abundance of others.

Metabolomics is an analytical profiling approach for measuring all metabolites in a given organism or biological sample [44]. Multi-dimensional LC-MS/MS combined with multi-label and no-label analysis has been widely used for the comparison of metabolites [45]. Here, we measured metabolites in a fermentation system with different concentrations of OA by LC-MS/MS. Corresponding to the beta-diversity results of the bacterial community, OA exposure had a limited effect on the overall composition of metabolites (Figure 4). However, compared to the control group, lots of differential metabolites were observed in the 50–1000 nM OA groups, including 31 shared differential metabolites. Only 4 of the 31 shared metabolites matched explicit annotated information in the secondary MS results, and four metabolites were absent in the control group, including OA and DTX-2. According to OA concentrations and the abundance of metabolites in different groups, we screened out the other five differential metabolites absent from the control group beyond the 31 shared differential metabolites. They were also shown to possess a strong correlation with OA metabolism, as proved by the Pearson test. Though lespedezafavanone F and tolytoxin detected in vivo were not detected in the fermentation system [25], the detection of DTX-2, POS-827.46, POS-769.46, POS-828.46, NEG-804.46, POS-770.46, POS-752.45 and POS-751.45 provided some evidence that special gut bacteria could take part in the metabolism process of OA. However, the contribution of bacteria in the overall OA metabolism process needs to be further explored, which is of significance for revealing the complex toxicity of OA.

Further, we analyzed the bacteria that may be involved in OA metabolism through the correlation between abundance of the 25 significant affected bacterial genera and the main metabolites of OA. Genera of *Faecalitalea*, *Lachnoclostridium*, *Butyricimonas* and *Roseburia* presented a correlation with the metabolic process of OA to some extent, especially *Faecalitalea*. *Faecalitalea* belongs to the Firmicutes, which can ferment D-glucose, sucrose, D-mannose and raffinose, and the main end product of metabolism is butyric acid [46]. The strong correlation with OA metabolism makes it a potential OA-degrading bacterium. Furthermore, *Faecalitalea* might protect intestinal barrier function by producing short-chain fatty acids [47], which also makes it a potential beneficial bacterium for the clinical treatment of DSP. However, we did not find a correlation between *Bacteroides* associated with OA metabolism in vivo and OA metabolism in fermentation systems [25]. It is worth noting that there are huge differences in conditions between in vitro and in vivo, and the metabolic processes are multiple and varied. In addition, our study has other limitations, such as the use of GAM medium and a single 24 h in vitro fermentation. Given the differences in gut microbiota between rats and humans, we focused more on bacteria commonly found in different hosts in this study. Even so, the direct transferal of the results obtained in rats to humans is not rigorous. The improvement of fermentation process is urgent for further study on in vitro metabolism of OA. Nevertheless, screening specific OA-degrading bacteria to cope with increasing contamination of shellfish has certain prospects.

## 4. Materials and Methods

### 4.1. Material

Okadaic acid (purity ≥ 98%) was purchased from LC laboratories (Woburn, MA, USA), and dissolved in normal saline solution (0.9% NaCl) containing 1.8% ethanol (*v*/*v*) with concentration of 100 μg/mL before use. Different proportions of the working solution were then mixed with solvent to form a series of solutions with final concentrations of 0–1000 nM. Female Wistar rats (10 weeks) were provided by SPF Biotechnology Co., Ltd. (Beijing, China). GAM, vitamin K1 and hemin were purchased from Hopebiol (Qingdao, China).

### 4.2. Collection of Gut Microbiota

This work was reviewed and approved by Institutional Animal Care and Use Committee (IACUC) of Jinan University (Approval No. IACUC-20211027-01). A total of 12 animals were randomly assigned to four cages and fed freely every day under the 12 h light/12 h dark cycle, 23 ± 2 °C, the humidity 50–70%. After 10 days of quarantine, freshly excreted feces were collected from each animal immediately, avoiding contamination, and mixed well with sterilized modified normal saline solution (cysteine-HCl 0.5 g/L and NaCl 9.0 g/L) to obtain 10% (*w*/*v*) fecal suspension [48]. After centrifugation at 300× *g* at 4 °C for 5 min, the supernatant and sediment containing gut microbiota were separated and used for in vitro fermentation immediately.

### 4.3. Fecal Microbial Culture Fermentation

Fecal microbial culture fermentation was carried out according to previous studies with some modifications [49]. After fully dissolving in ddH_2_O, GAM was adjusted to pH 7.0, autoclaved (121 °C, 210 kPa) for 30 min, and transferred to an anaerobic incubator YQX-II (Yuejin, Shanghai, China). Before use, sterile 0.1% vitamin K1 and sterile hemin (final concentration at 5 mg/L) were added to the medium in a ratio of 1:1000 (*v*/*v*). The collected supernatant was inoculated in the fermentation medium at a volume ratio of 1:20. Given that bacteria are thought to be more tolerant than cell lines due to the cell wall, the final OA concentrations of 0, 50, 100, 250 and 1000 nM were set to verify the direct interaction of OA and gut microbiota according to the cytotoxicity of OA on cell lines [10,32,33,34]. The fermentation system was cultured at 37 °C with 200 rpm (amplitude, 20 mm) under an anaerobic environment for 24 h. After centrifugation at 4000× *g* for 10 min at 4 °C, and the microbiota and supernatant were separated and stored at −80 °C until further analysis.

### 4.4. Structure Analysis of Bacterial Community

Total DNA of cultured gut microbiota in GAM with different concentrations of OA were extracted with PowerSoil^®^ DNA Isolation Kits (MOBIO Laboratories, Carlsbad, CA, USA) and quantified by using a Nano Drop One (Thermo Fisher Scientific, Waltham, MA, USA). The V3–V4 regions of bacterial 16S rDNA were amplified by using the specific primers 338F (5′-ACTCCTACGGGAGGCAGCA-3′) and 806R (5′-GGACTACHVGGGTWTCTAAT-3′) [50]. PCR was performed on BioRad S1000 (Bio-Rad Laboratory, Hercules, CA, USA) with the following conditions: 5 min at 94 °C; 30 cycles of 30 s at 94 °C, 30 s at 52 °C, and 30 s at 72 °C; followed by 10 min at 72 °C. PCR products were purified by an EZNA Gel Extraction kit (Omega Bio-Tek, Norcross, GA, USA) after running gel-electrophoresis and used to generate the sequencing libraries.

Sequencing was carried out on an Illumina-Hiseq 2500 platform. Raw reads were filtered by using Trimmomatic v0.33, merged with FLASH v1.2.11, and quality controlled with Mothur v.1.35.1 [51,52,53]. Operational taxonomic units (OTUs) were picked at 97% similarity cutoff by USEARCH v10 after removing chimeras and singletons [54]. Representative OTU sequences were assigned to the SILVA database (SILVA, Available online: http://www.arb-silva.de, accessed on 20 January 2022) to annotate taxonomic information using ribosomal database project (RDP) by QIIME v1.9.1 with default parameters [55,56]. Species diversity was analyzed using alpha diversity based on the observed OTUs, and beta-diversity was estimated by calculating Bray–Curtis dissimilarity between samples.

### 4.5. Extraction of Metabolites from Fermentation System

Fermentation supernatant sample from each fermentation system were thawed at 4 °C. Then, 100 mL of fermentation supernatant was blended with 400 μL of methanol–acetonitrile solution (1:1, containing isotope-labeled internal standard mixture), and followed by vortexing for 30 s. After 10 min of sonication in an ice-water bath, the suspension was incubated at −40 °C for 1 h to precipitate proteins, then centrifuged at 13,800× *g* for 15 min at 4 °C. The resulting supernatants was collected and transferred to LC-MS vials stored at −80 °C for LC-MS/MS analysis.

### 4.6. LC-MS/MS Analysis

The supernatant samples were randomly injected for LC-MS/MS analysis. A quality control (QC) sample was prepared by mixing equal aliquots of the supernatants from all of the samples and used for data normalization. Blank samples and QC samples were injected every five sample during acquisition.

LC-MS analysis was performed using an UHPLC system (Vanquish, Thermo Fisher Scientific, Waltham, MA, USA) with an HPLC BEH Amide column (2.1 mm × 100 mm, 1.7 μm) coupled to quadrupole-Orbitrap (Q-Exactive) HFX mass spectrometer (Orbitrap MS, Thermo Fisher Scientific, Waltham, MA, USA). The auto-sampler temperature was 4 °C and the injection volume was 2 μL. The mobile phase consisted of A (25 mmol/L ammonium acetate and 25 mmol/L ammonia hydroxide in water, pH = 9.75) and B (acetonitrile). MS/MS spectra were acquired by using a QE HFX mass spectrometer on information-dependent acquisition (IDA) mode in the control of the acquisition software (Xcalibur, Thermo Fisher Scientific, Waltham, MA, USA). The ESI source conditions were set as follows: sheath gas rate, 30 Arb; auxiliary gas flow rate, 25 Arb; capillary temperature, 350 °C; full MS resolution, 60,000; MS/MS resolution, 7500; collision energy, 10/30/60 in NCE mode; spray voltage, 3.6 kV for positive or −3.2 kV for negative, respectively.

The raw data were converted to the mzXML using ProteoWizard and processed with R based on XCMS for peak detection, extraction, alignment, and integration, as reported in previous studies [57,58]. The preprocessing generated a data matrix that consisted of the retention time (RT), mass-to-charge ratio (m/z) values, and peak intensity. The resulting matrix was further managed by removing noise based on relative standard deviation (RSD) and removing peaks with any missing value (ion intensity = 0) in more than 50% of the samples [59]. The missing values in the obtained data were simulated, and half of the minimum value was used to recode the missing value. The RSD values of the QC sample were calculated, and the most stable internal standard was screened for the data normalization. The metabolites were identified using an in-house MS2 database named BiotreeDB (v2.1) with a cutoff of 0.3 [60], and matched to the public databases including the Human Metabolome Database (HMDB), PubChem and KEGG (see details in Supplementary Materials).

### 4.7. Statistical Analysis

Data were analyzed using GraphPad prism 7.00 software (GraphPad Prism, Inc., San Diego, CA, USA), and presented as the mean ± standard error (SE). Statistical comparisons of continuous variables in accordance with normal distributions were calculated by using Student’s *t* test or ANOVA followed by Tukey’s multiple comparison test. When the variances were not homogeneous, the data were normalized for analysis by Welch’s ANOVA followed by the Games-Howell method. Statistically significant differences are indicated as * *p* ˂ 0.05. Correlation analyses were performed using Pearson’s or Spearman’s correlation test. Correlations were classified in weak (0 < r < 0.30), moderate (0.30 ≤ r < 0.60), strong (0.60 ≤ r < 0.90) and very strong (0.90 ≤ r < 1).

## 5. Conclusions

In conclusion, OA had shaping ability on the diversity of the bacterial community in vitro. OA could affect *Rothia*, *Bacteroides*, *Prevotellaceae* Ga6A1 group, *Helicobacter*, GCA900066575, *Lacnchnospiraceae* FCS020 group, *Morganella* and *Proteus*, and promote *Lanchnoclostridium* and *Faecalitalea*. Some metabolites of OA, including DTX-2 and another seven unidentified products, were detected in fermentation culture. The abundance of *Faecalitalea*, *Lachnoclostridium*, *Butyricimonas* and *Roseburia* showed correlation with OA metabolism, especially *Faecalitalea*. Our findings provide evidence for the interaction between OA and gut bacteria, which is helpful to reveal the metabolic process of OA and elucidate the complex toxicity of OA.

## Figures and Tables

**Table 1:** Dissimilarity test between OA-exposed groups and control group (0 nM) based on Bray–Curtis distance (*n* = 3).

Dissimilarity Test (Compared with 0 nM)	Adonis		Amova	
	R2	*p*-Value	Fs	*p*-Value
50 nM	0.315	0.200	0.033	0.182
100 nM	0.036	0.900	0.006	0.919
250 nM	0.306	0.200	0.028	0.214
1000 nM	0.303	0.100	0.027	0.099

**Table 2:** Differential metabolites in the OA-exposed groups compared to the control.

Ion Mode -m/z	MS2 Name	Average Peak Area				
		0 nM	50 nM	100 nM	250 nM	1000 nM
POS-847.4		772,914.5	1,354,450.7	1,245,177.5	1,238,153.1	1,361,723.0
POS-827.46		0.0	64,009.6	244,445.6	779,112.4	4,457,365.8
NEG-803.46	OA	0.0	817,356.1	1,384,218.9	4,597,209.1	21,190,896.5
POS-787.47	DTX-2	0.0	71,343.8	235,165.0	1,146,066.1	5,208,008.2
POS-769.46		0.0	108,862.8	431,789.1	1,489,167.6	6,906,719.2
POS-665.5		214,218.8	559,356.9	637,709.5	730,160.9	806,568.0
POS-492.86		625,484.9	904,944.3	817,592.2	873,855.3	879,275.2
POS-429.14		26,078.9	650,092.3	1,050,385.8	1,424,048.2	1,468,577.4
POS-422.15		222,449.8	625,832.1	647,563.7	853,352.8	1,167,906.2
POS-351.12		259,230.5	720,817.2	681,464.2	845,456.2	642,511.6
NEG-343.25	Streptidine 6-phosphate	1,695,420.0	341,608.0	3,286,003.0	4,142,678.0	4,398,131.0
POS-331.25		9,802,187.2	18,301,392.0	16,095,423.7	24,730,228.0	23,451,383.2
POS-302.23		364,045.6	764,114.8	685,071.7	594,568.3	635,510.6
POS-286.01		1,765,617.3	4,276,114.8	5,063,965.6	6,421,677.2	7,146,981.4
POS-239.12		3,614,630.5	6,147,948.6	6,369,886.7	7,900,067.9	8,214,998.3
POS-238.12		48,583,494.9	73,257,098.7	76,581,091.2	96,541,315.3	96,654,785.7
POS-220.03		834,014.0	2,285,900.4	1,906,606.8	1,964,003.2	1,862,868.0
POS-198.04		15,112,106.4	23,673,256.5	25,004,665.9	23,302,260.8	26,790,744.5
NEG-190.11		13,633,947.1	22,980,736.4	24,228,301.7	26,926,848.1	28,425,445.8
NEG-184.07	Phosphorylcholine	18,489,454.0	18,368,797.2	16,243,717.3	16,906,562.7	15,754,843.3
NEG-182.03		35,467,400.7	22,823,567.8	26,780,550.4	23,895,222.5	26,635,669.7
NEG-176.1		33,279,171.4	51,094,329.5	53,722,869.8	65,596,077.6	79,499,673.8
NEG-176.09	2-minoheptanedioic acid	2,666,927.2	5,726,041.4	5,114,338.2	5,593,144.0	6,024,575.6
NEG-172.01		3,495,412.5	6,126,153.9	6,731,904.1	10,033,830.4	10,796,430.5
NEG-161.58		47,066.5	193,948.2	241,584.7	244,839.5	308,397.3
NEG-159.08		6,661,437.9	6,275,405.0	6,012,287.2	5,536,234.3	5,565,532.8
NEG-154.05		27,805,547.6	43,294,413.2	43,421,409.9	43,761,555.1	48,446,617.0
NEG-140.03		11,521,804.8	23,546,688.8	24,415,464.2	26,846,274.0	26,580,788.2
NEG-128.02		48,306,703.7	106,620,905.9	121,809,279.9	151,223,200.9	164,878,532.6
NEG-88.05		18,047,252.3	29,484,126.5	31,837,001.7	38,950,682.7	37,983,269.3
NEG-83.06		22,865,390.9	9,642,657.3	10,409,259.5	10,924,049.3	6,359,238.0

## Data Availability

The data in this study are available from the corresponding author upon request.

## Supplementary Materials

The following supporting information can be downloaded at: https://www.mdpi.com/article/10.3390/md20090556/s1, Figure S1: The Pearson correlation test among metabolites absent from the 0 nM group and the shared differential metabolites. Total ion chromatogram (TIC) diagram of positive ion mode detected by UHPLC-OE-MS; TIC diagram of negative ion mode detected by UHPLC-OE-MS; The matching index of all the identified compounds.
