# Supplementary material for: In Vitro Interactions between Okadaic Acid and Rat Gut Microbiome
Source: Mar Drugs. 2022 Aug 30;20(9):556. doi: 10.3390/md20090556 (PMC9500940; doi:10.3390/md20090556)
Supplement: Supplementary file 1 [file marinedrugs-20-00556-s001.zip › marinedrugs-1841172-supplementary/Supplement-Figure S1.pdf]

Figure S1

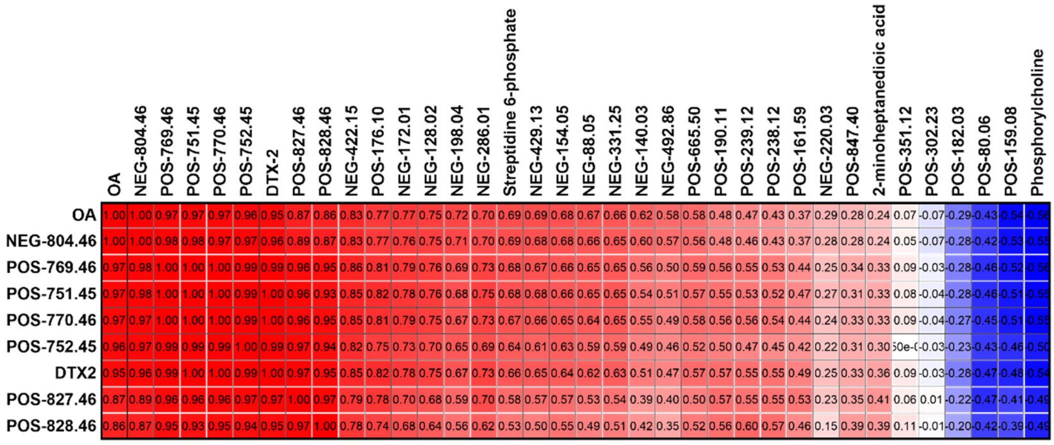

The Pearson correlation test among metabolites absent from the 0 nM group and the shared differential metabolites. The metabolites absent from the 0 nM group showed the most correlation with OA metabolism.
