# Supplementary figures and images for: In Vitro Interactions between Okadaic Acid and Rat Gut Microbiome
Source: Mar Drugs. 2022 Aug 30;20(9):556. doi: 10.3390/md20090556 (PMC9500940; doi:10.3390/md20090556)

1\_QC01 1\_QC02 1\_QC03

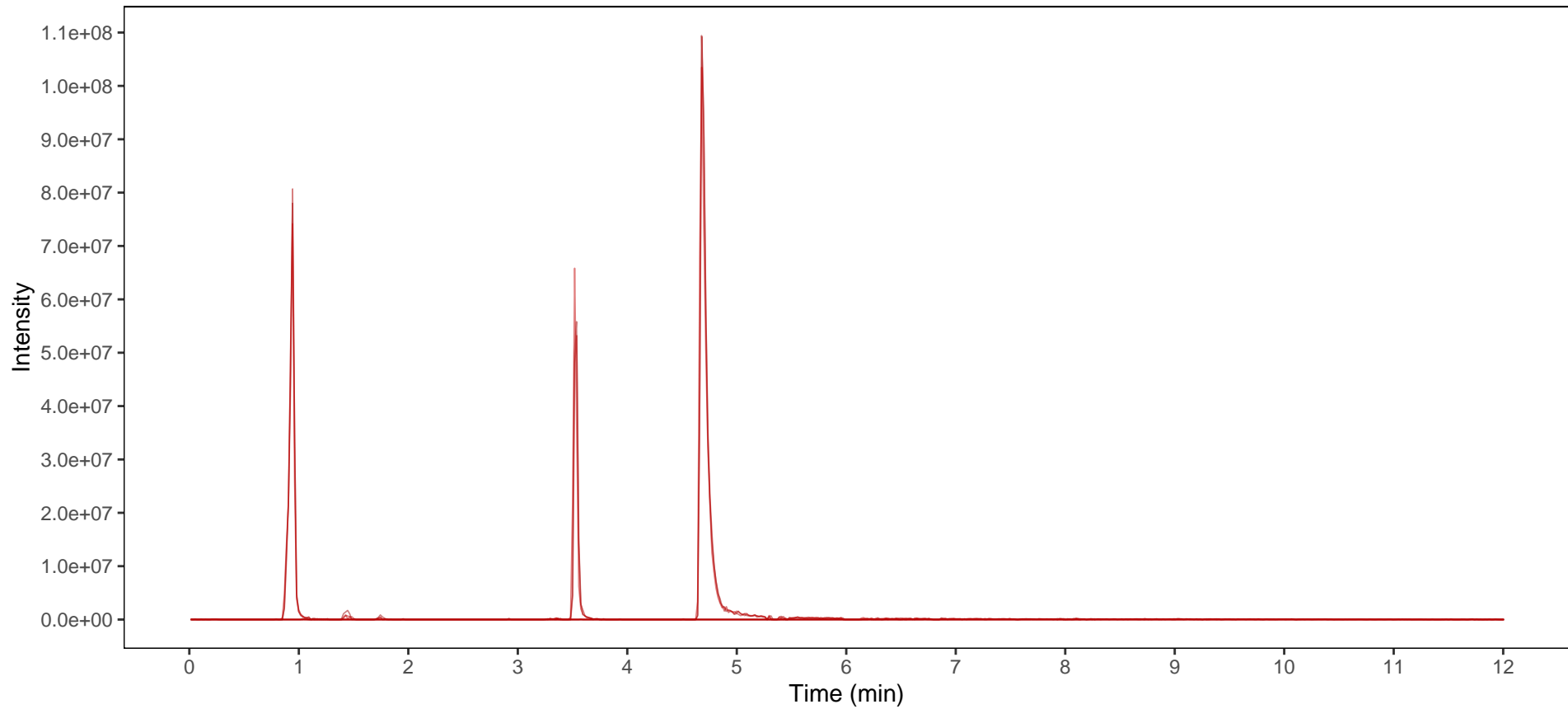

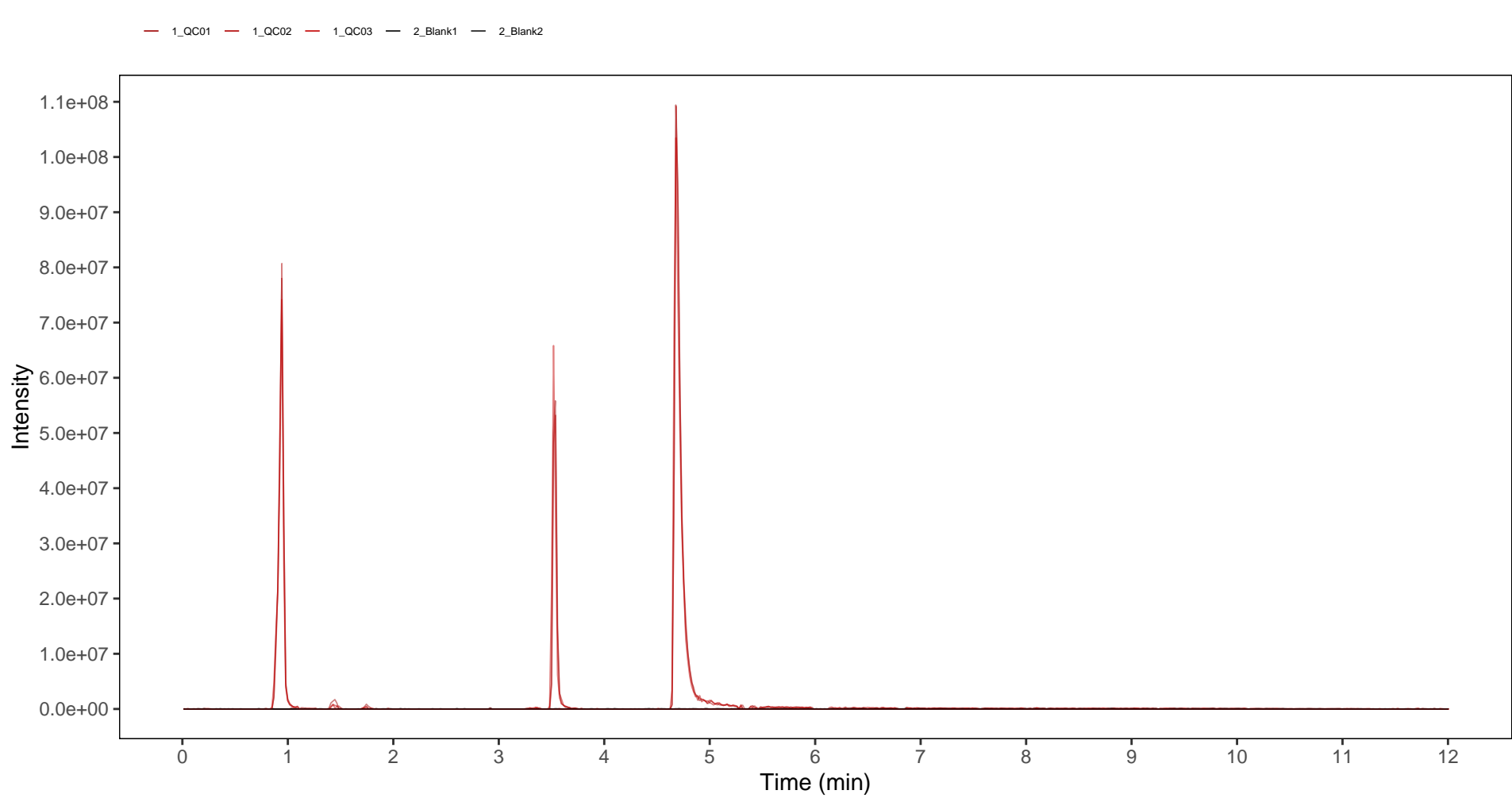

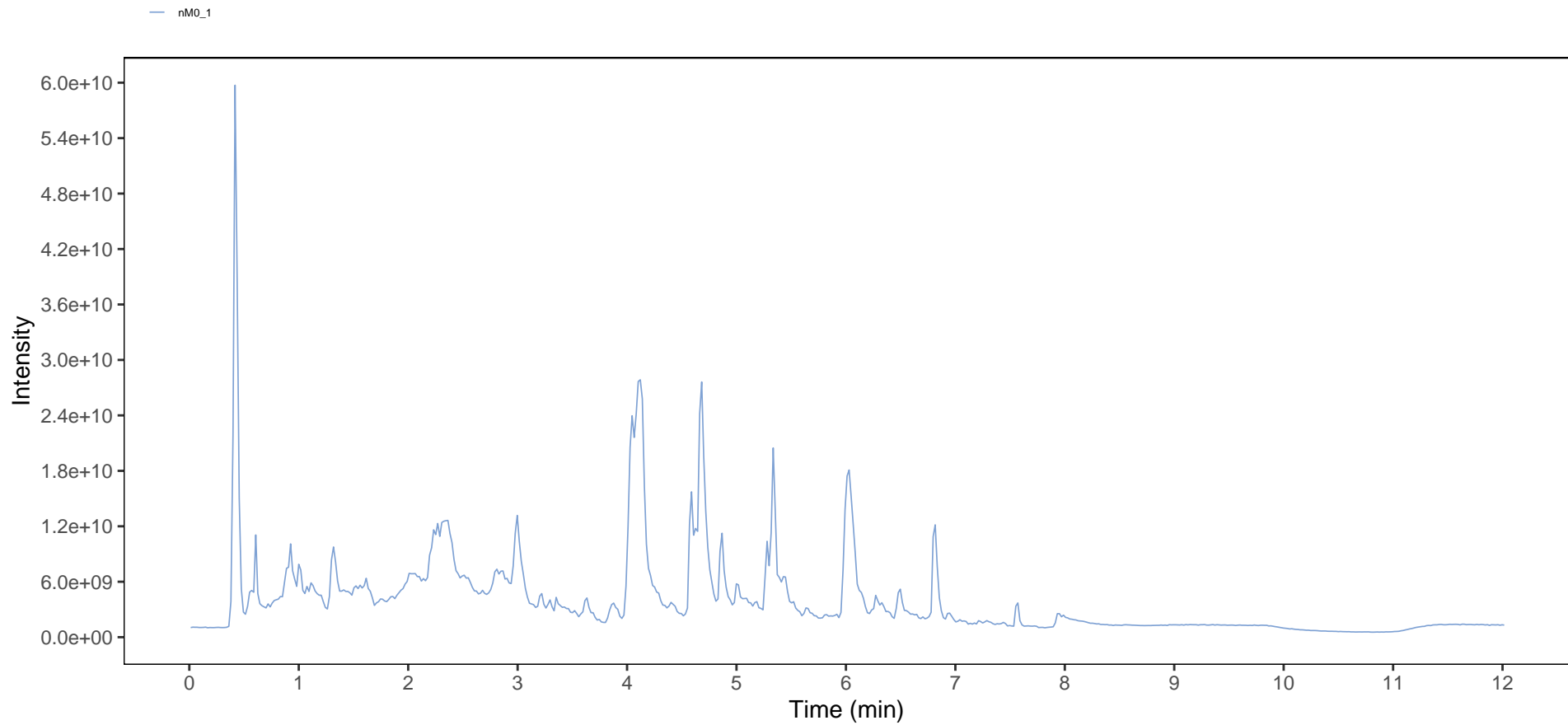

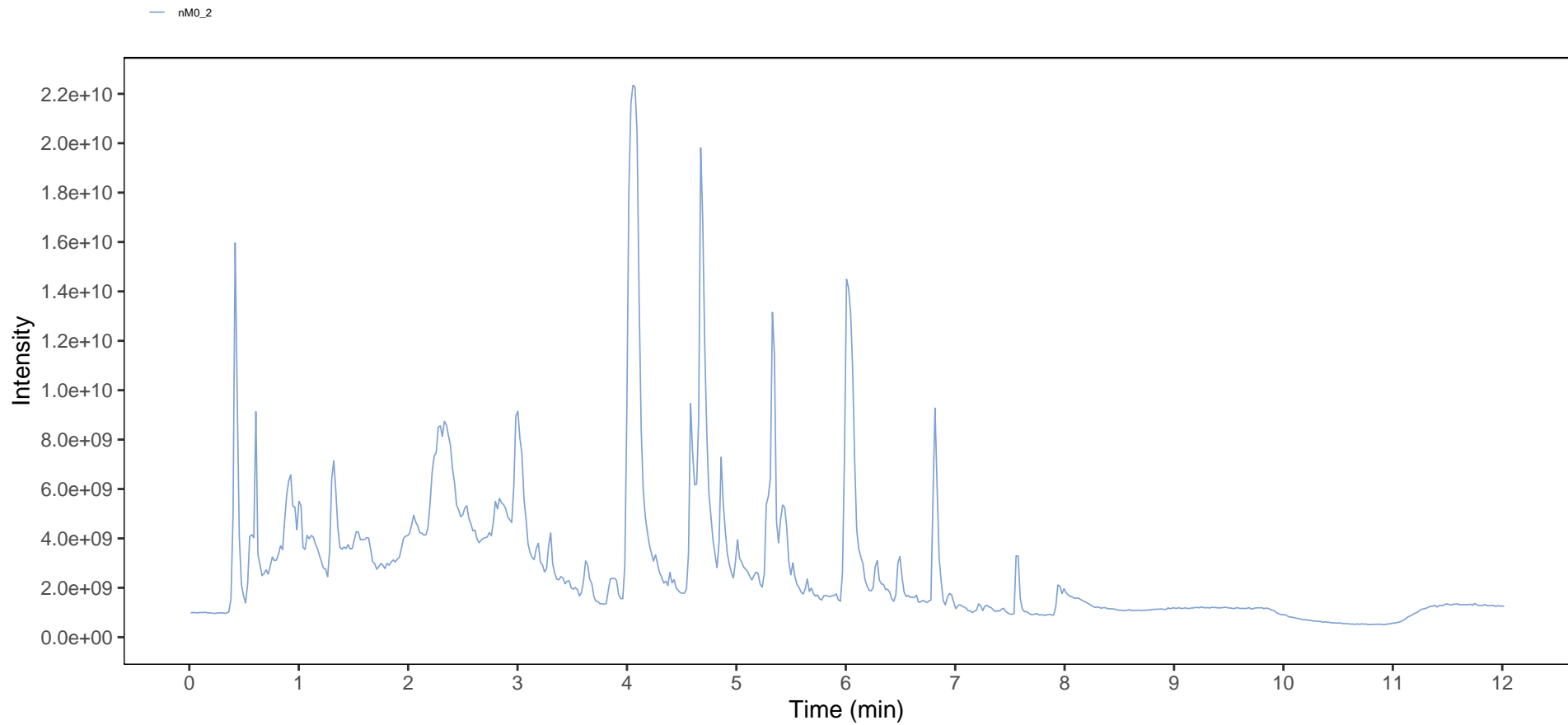

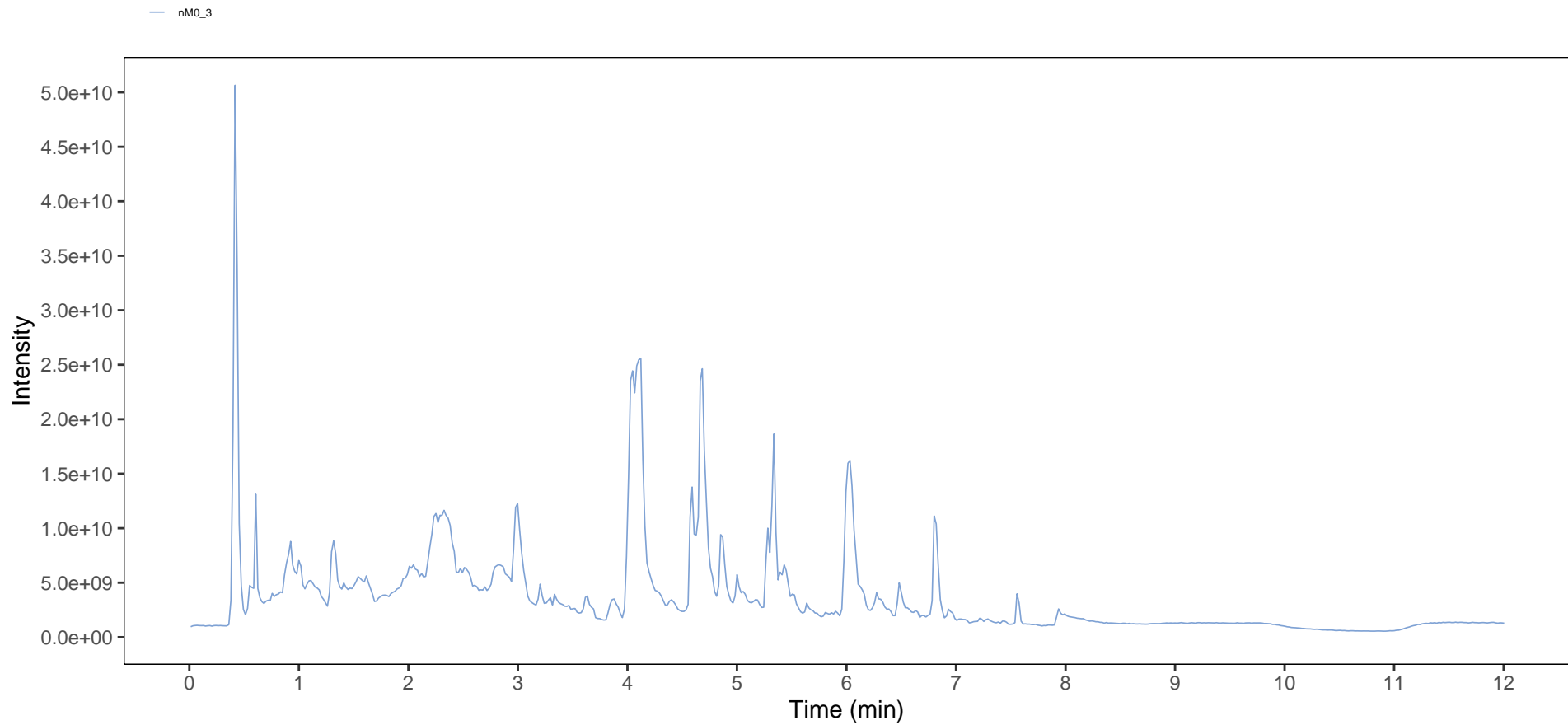

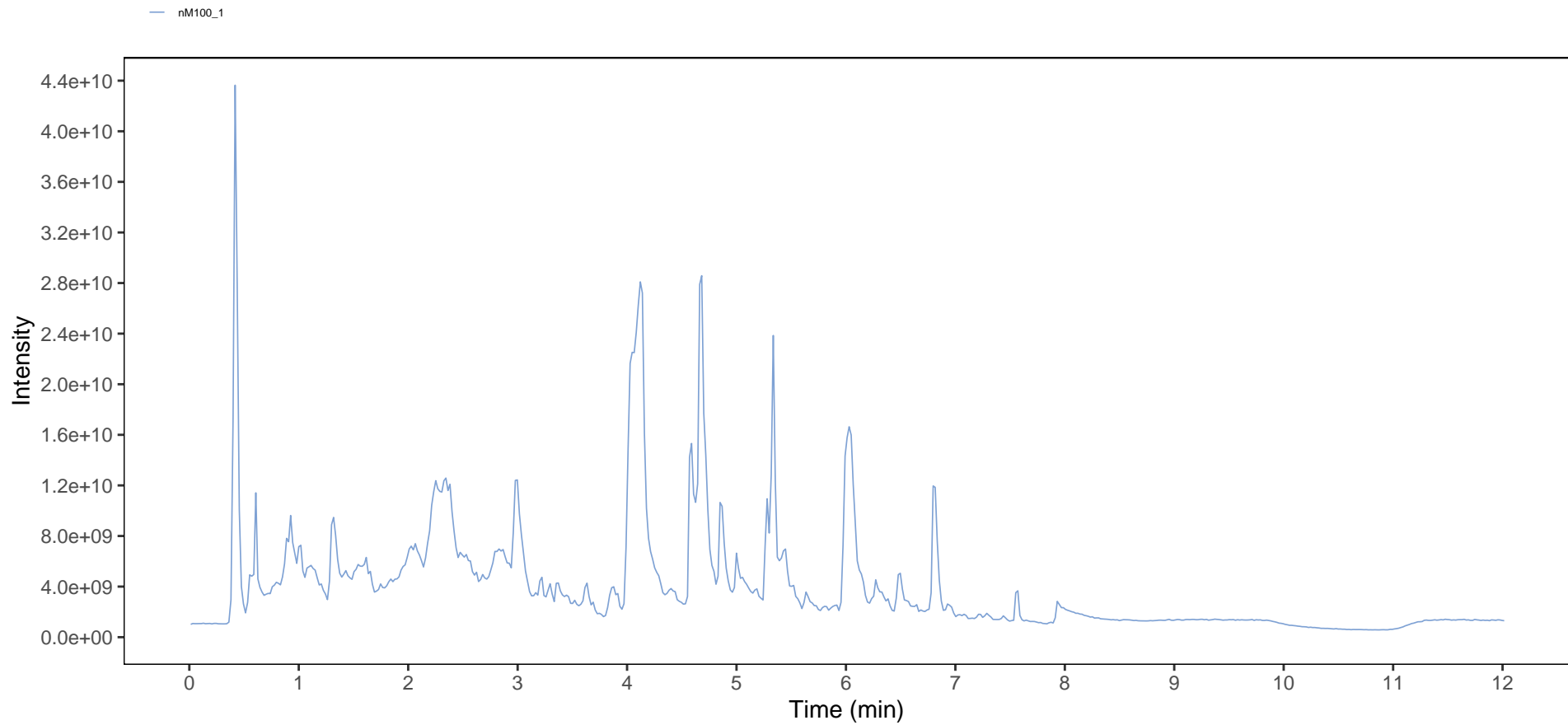

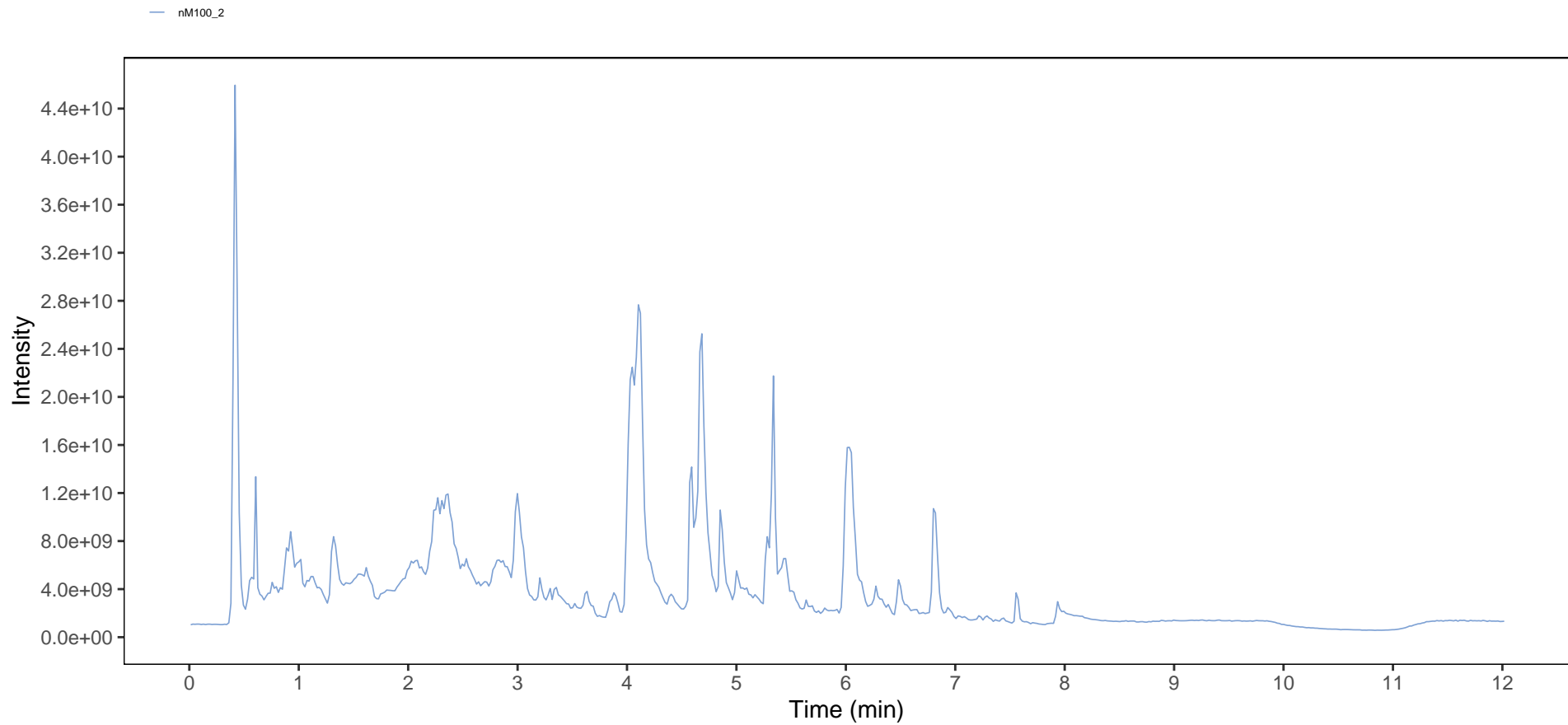

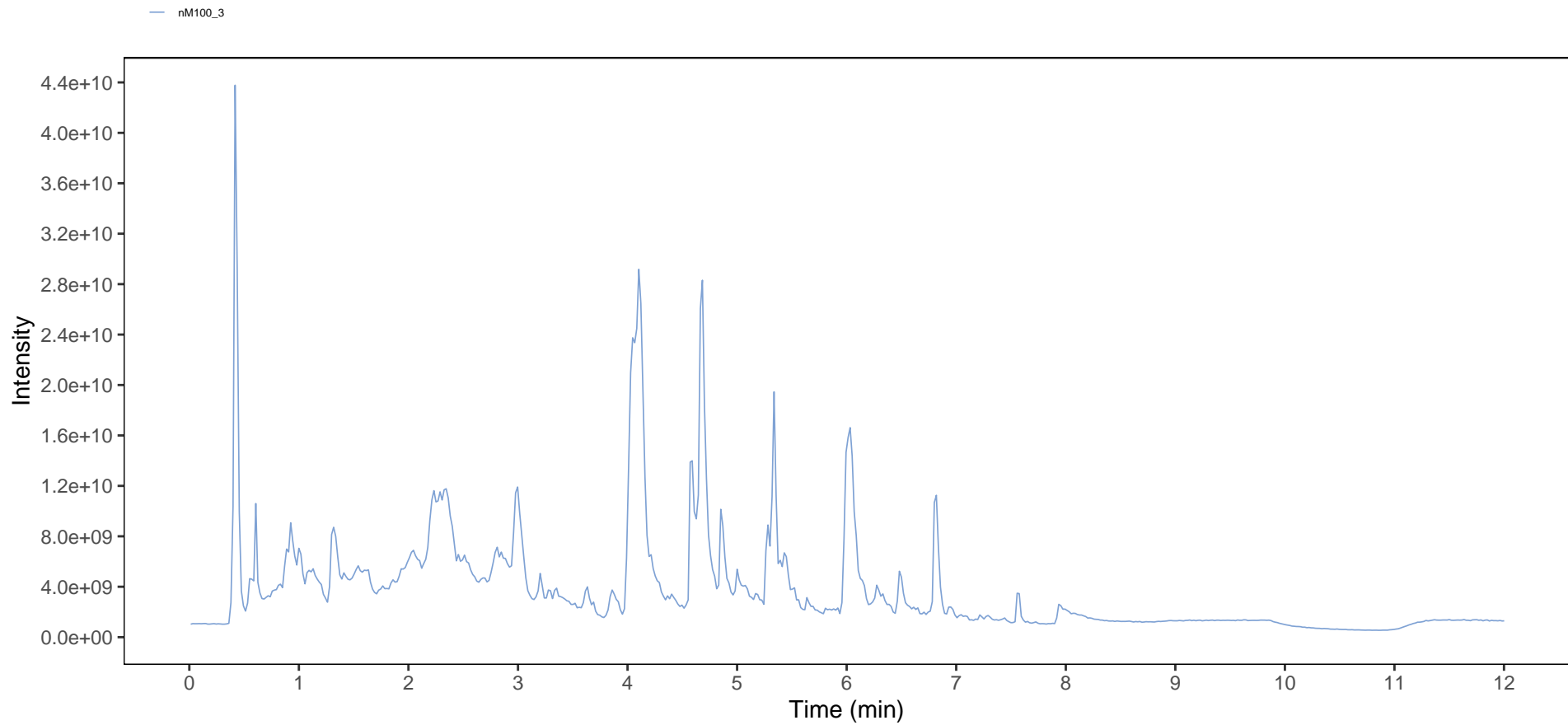

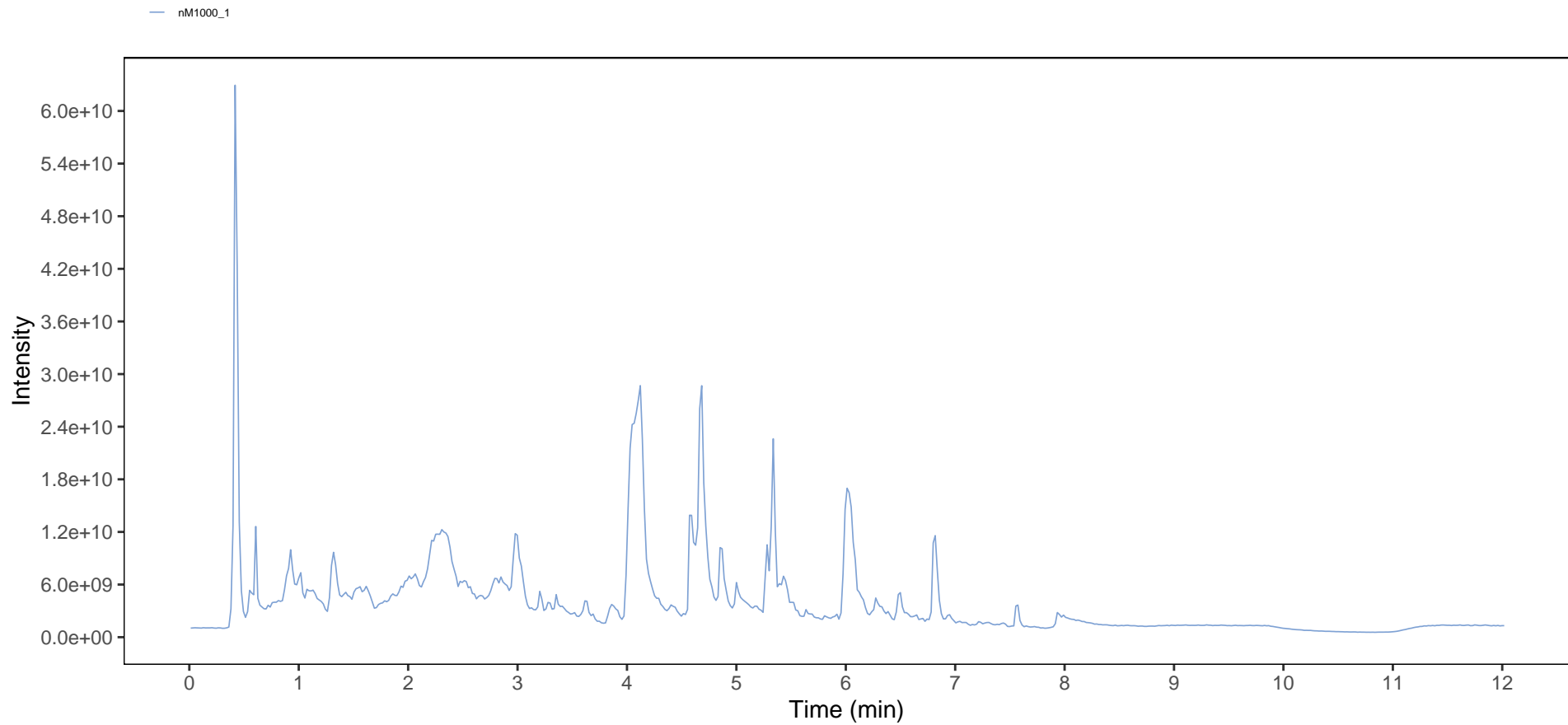

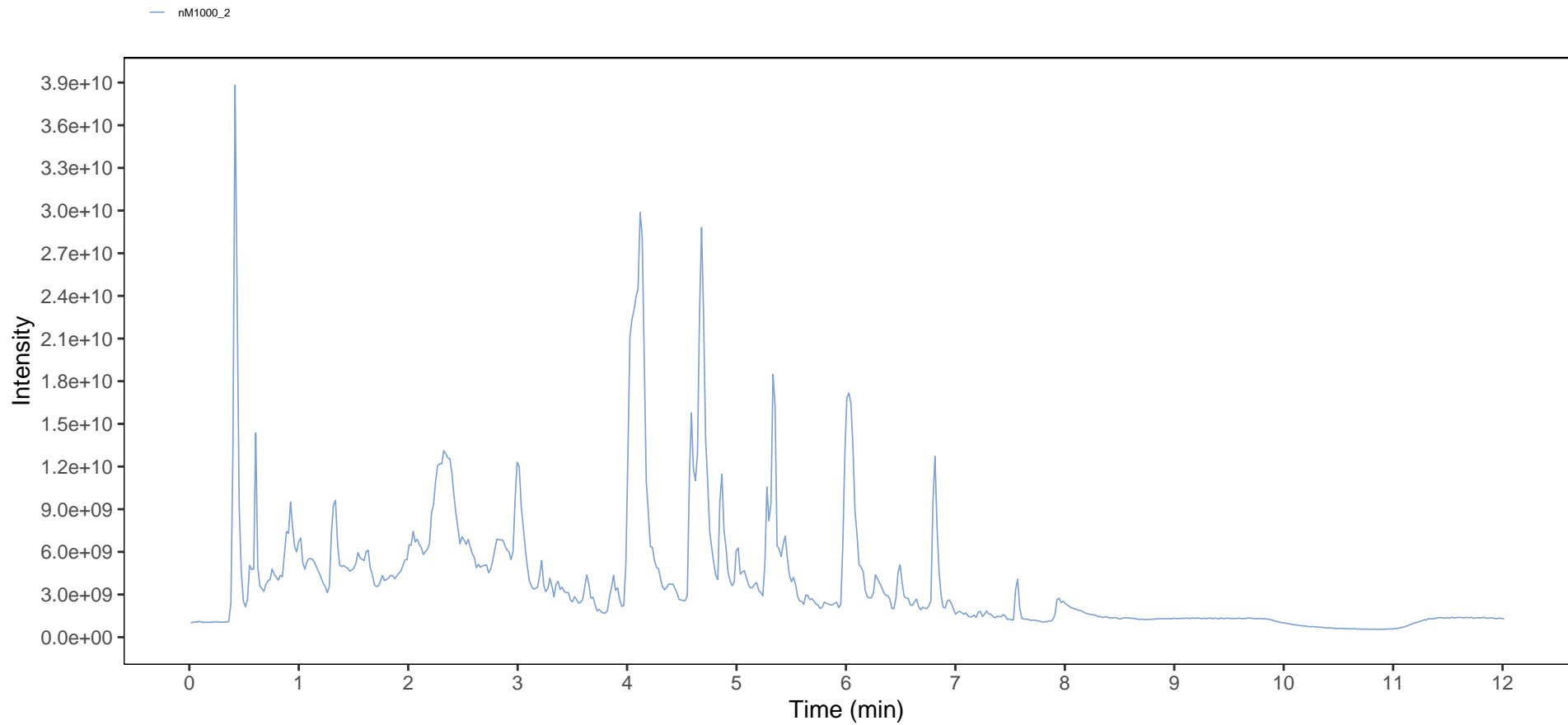

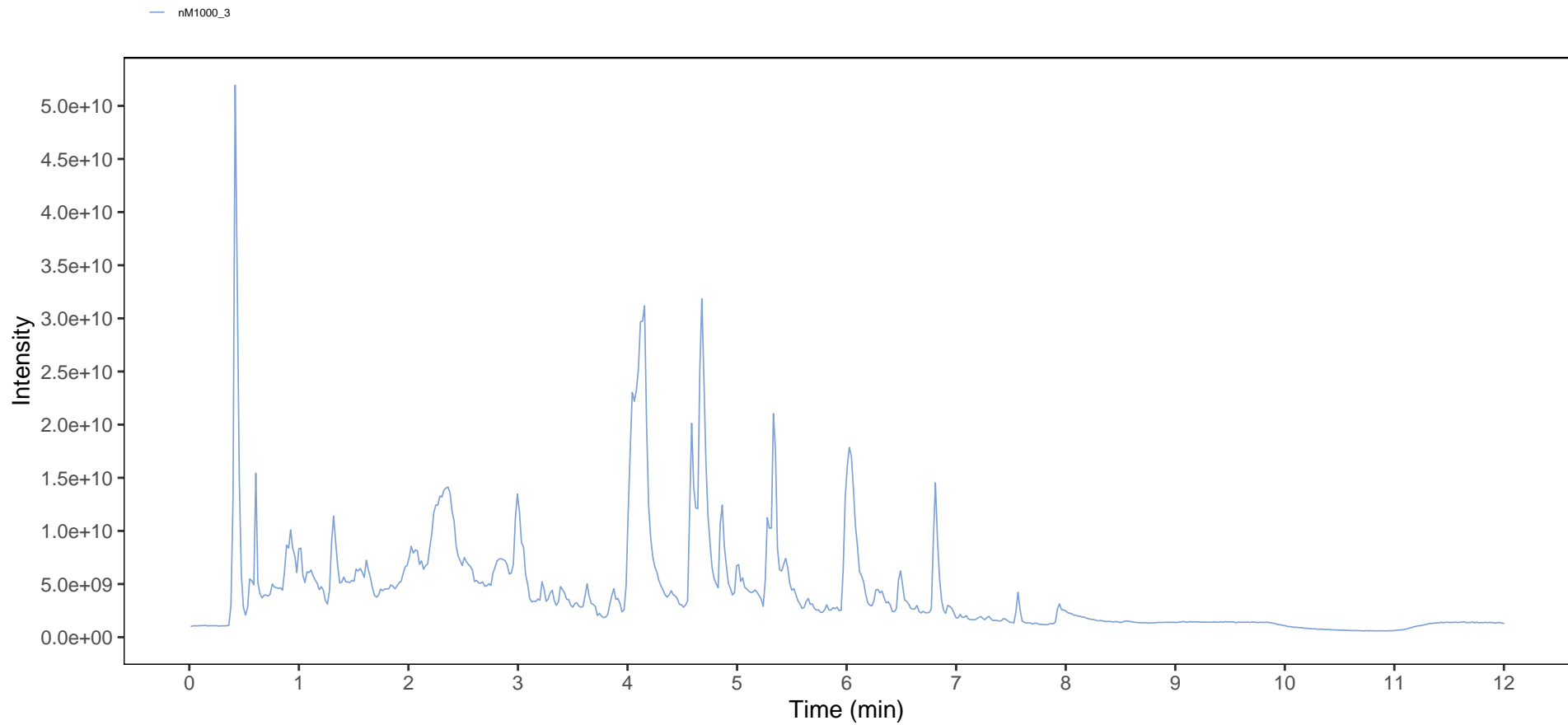

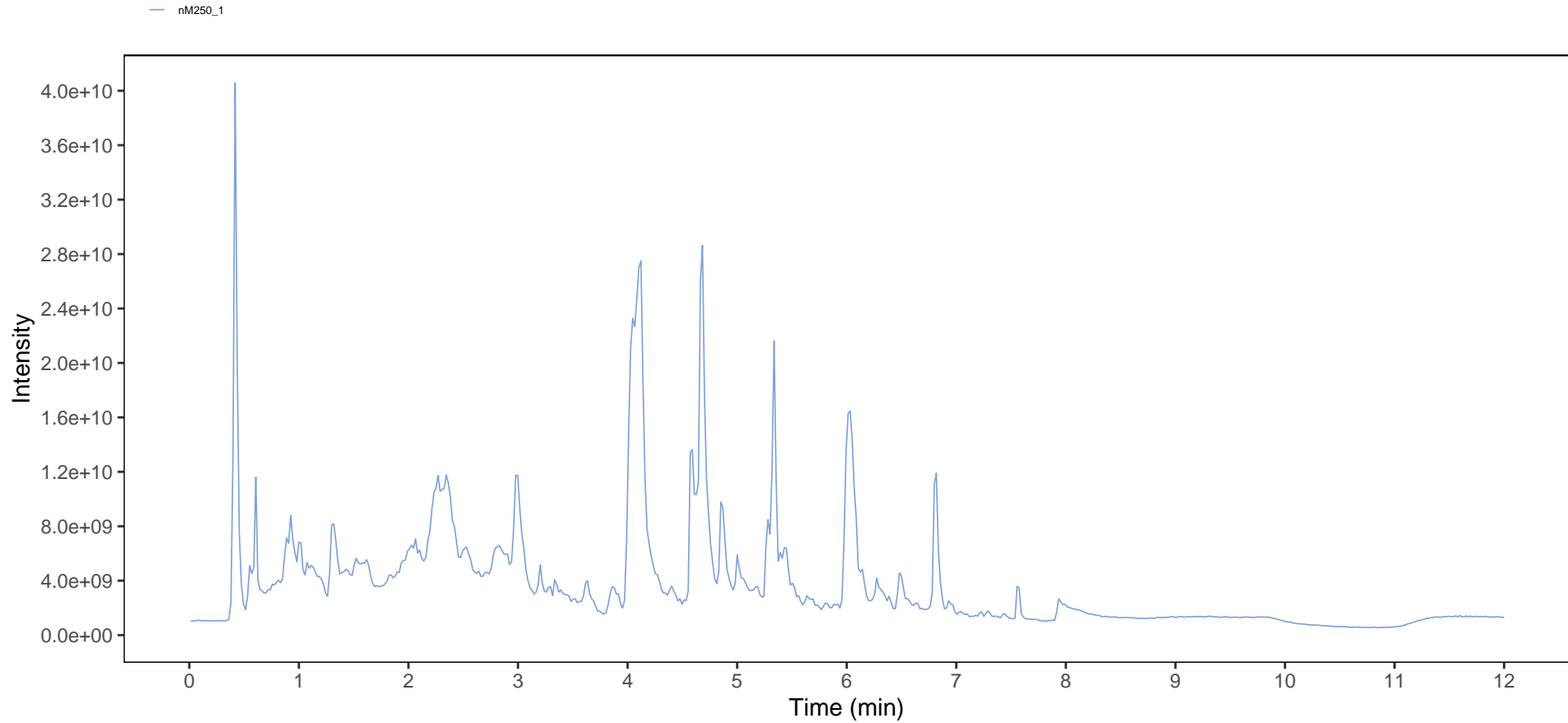

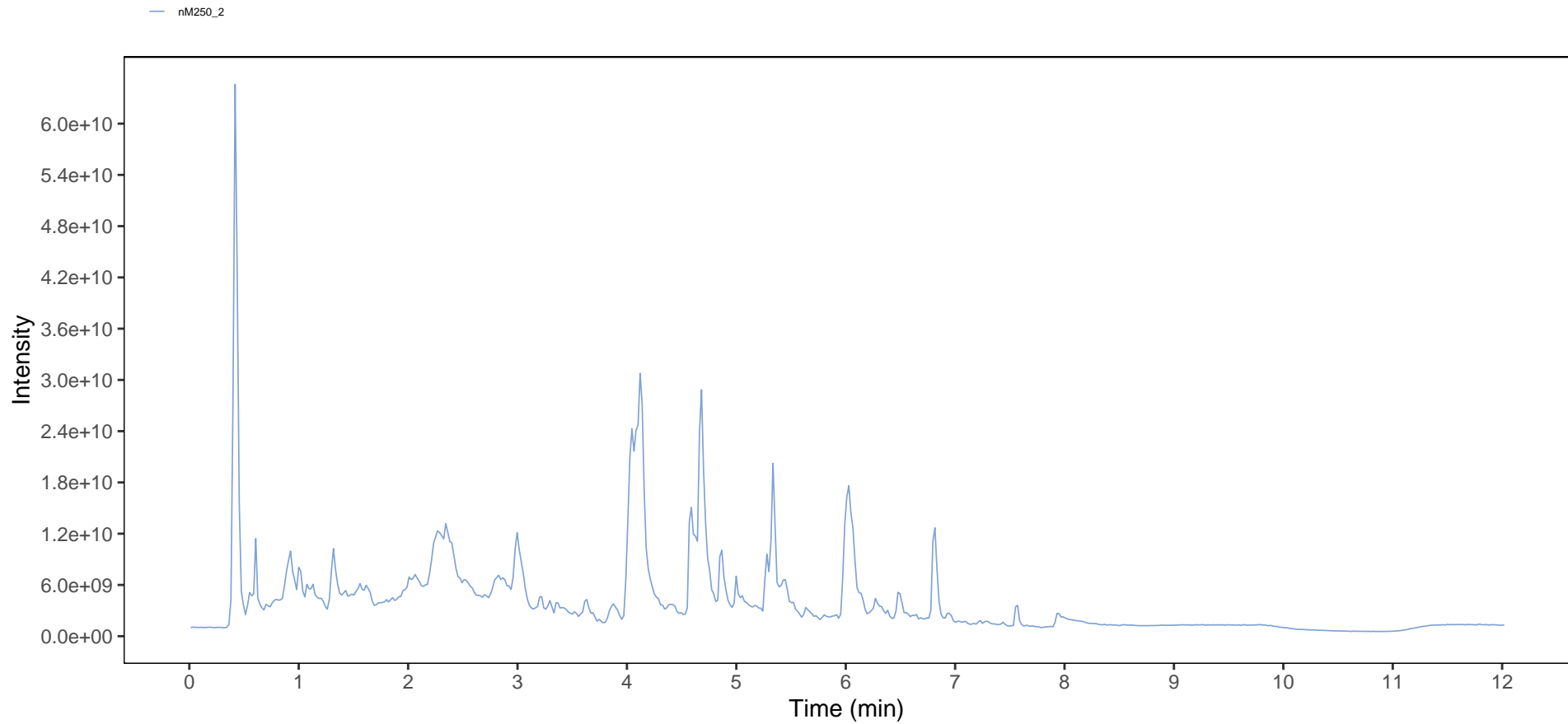

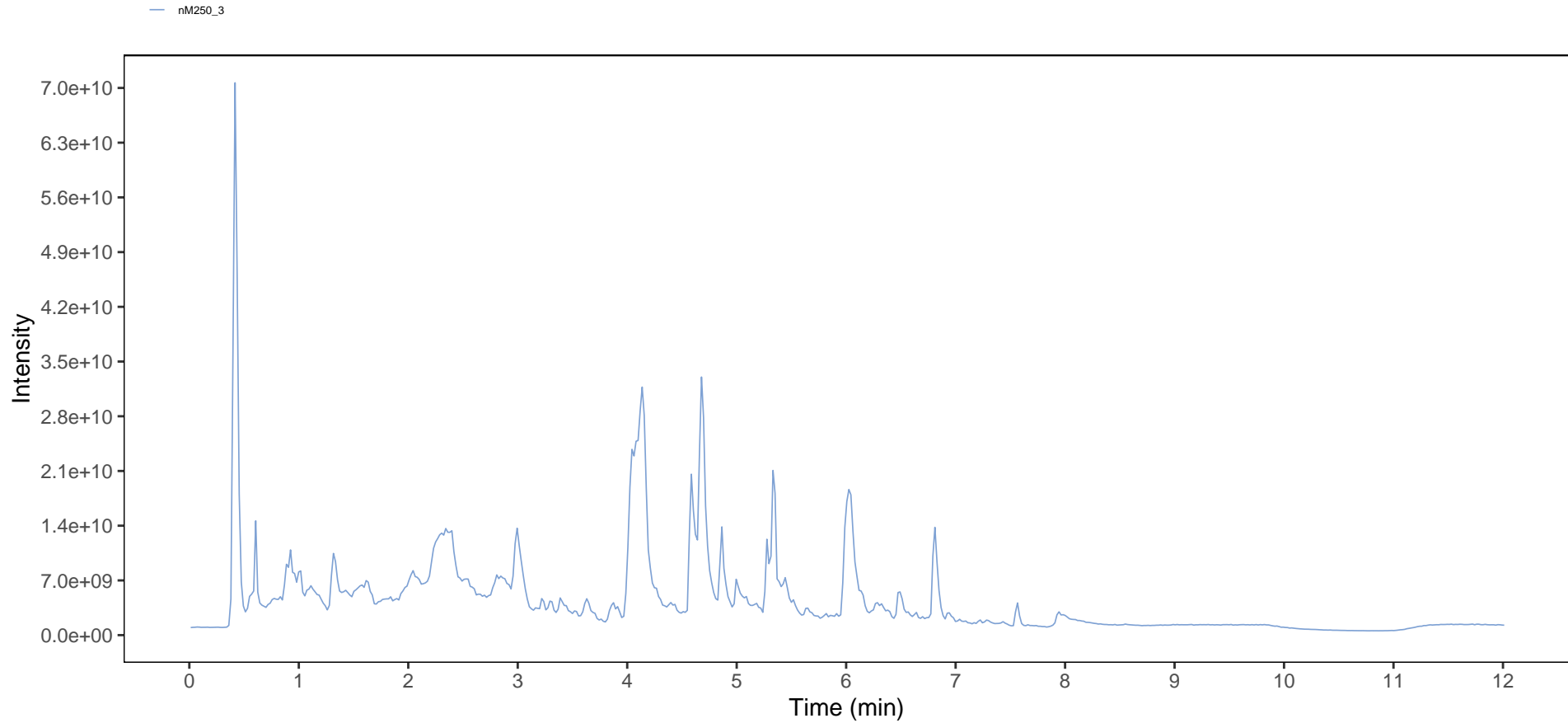

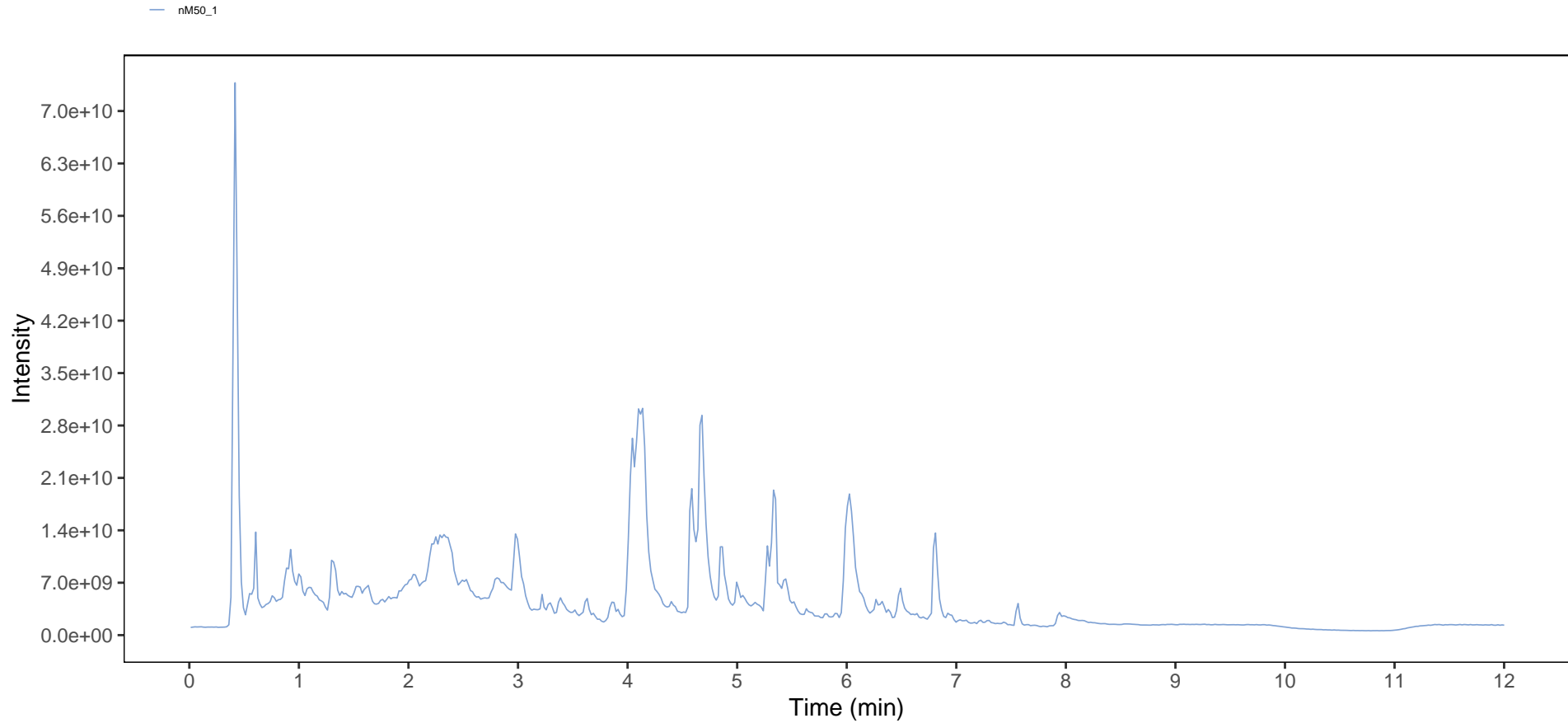

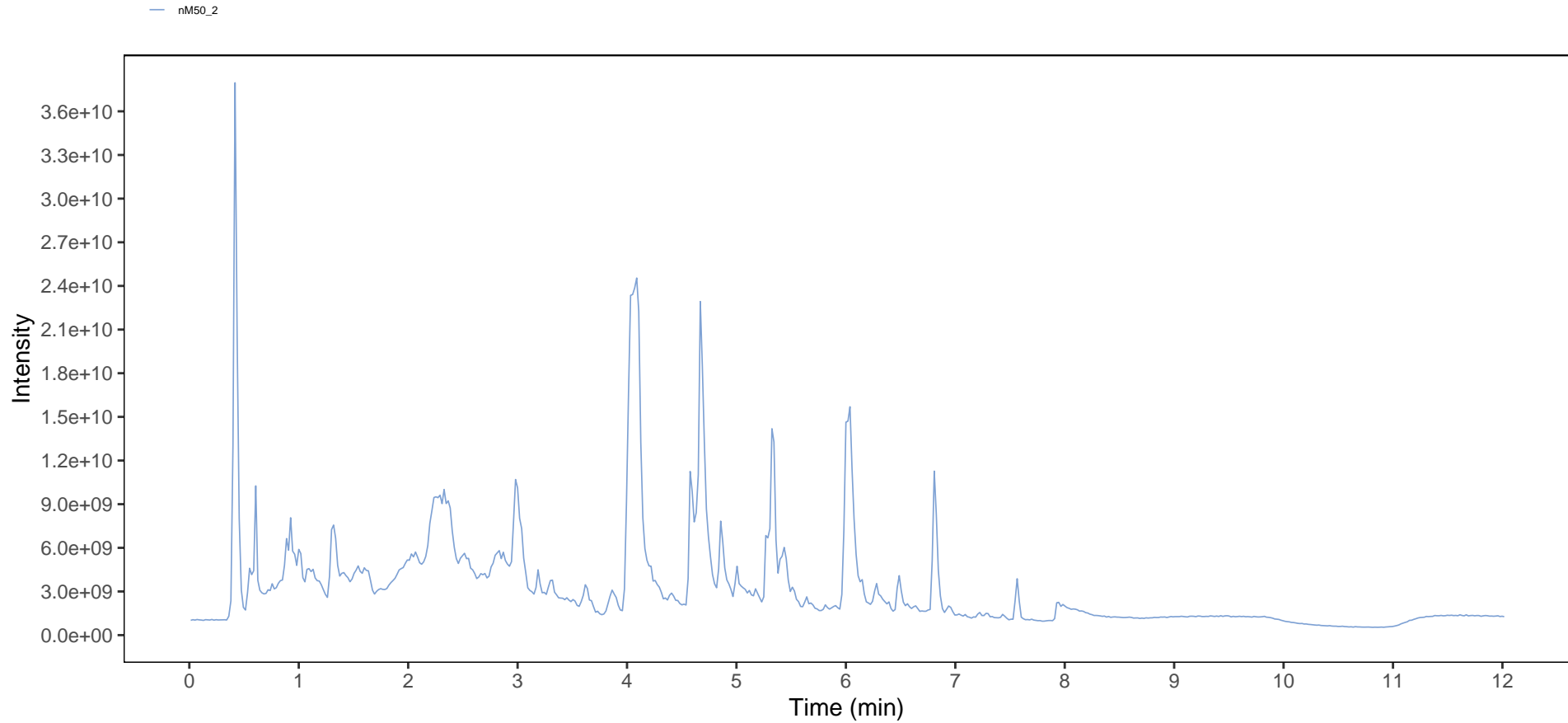

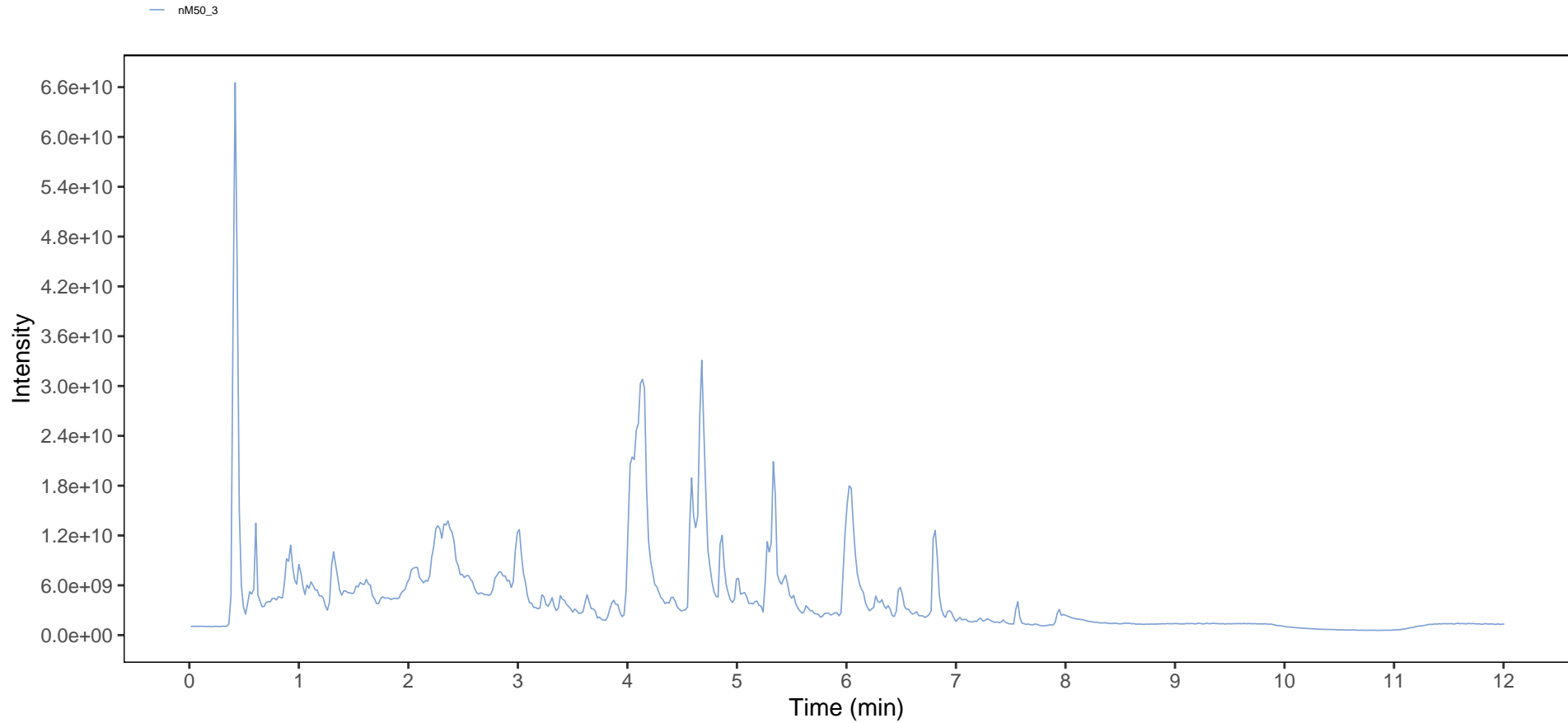

Supplement: Supplementary file 1 [file marinedrugs-20-00556-s001.zip › marinedrugs-1841172-supplementary/NEG-T.pdf]

1\_QC01 1\_QC02 1\_QC03

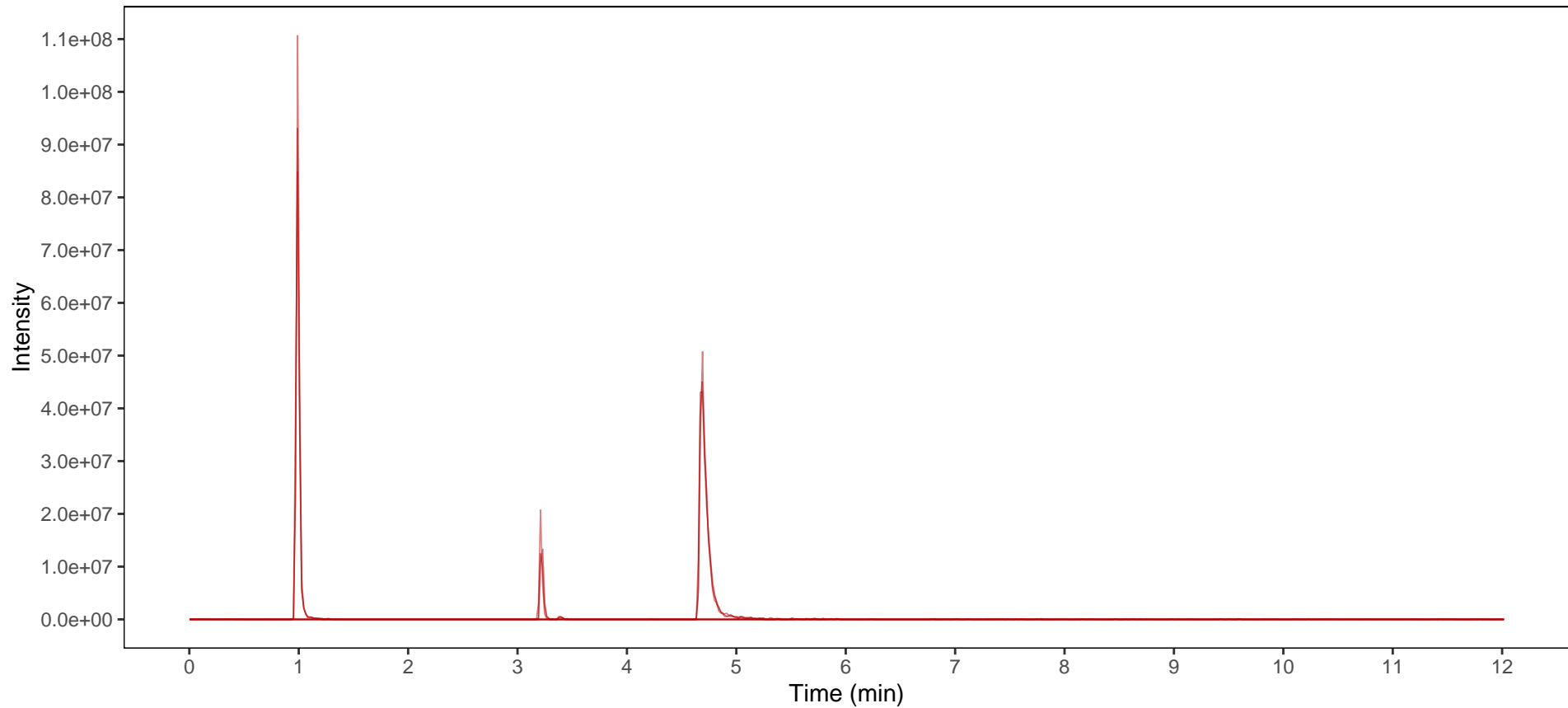

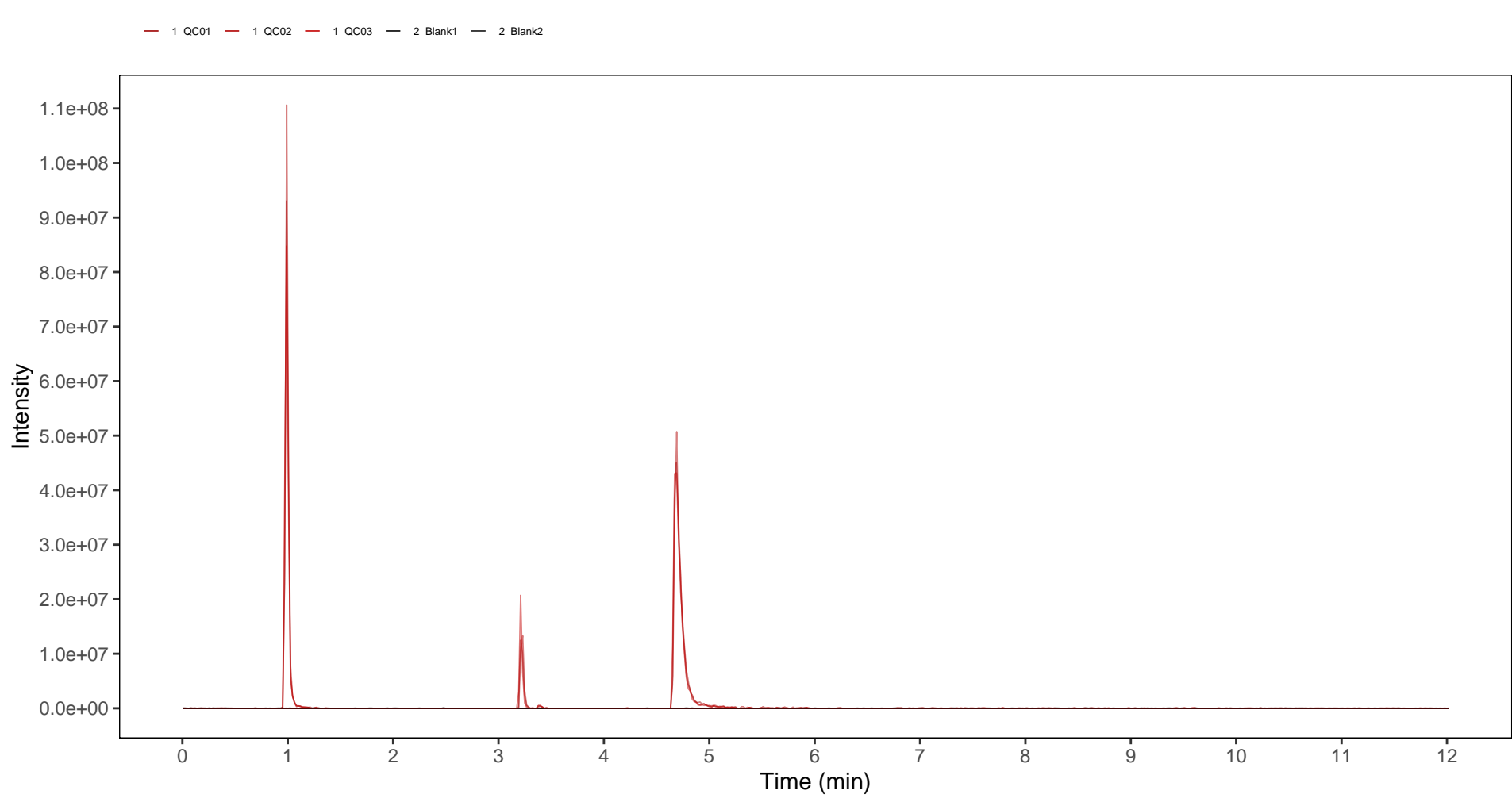

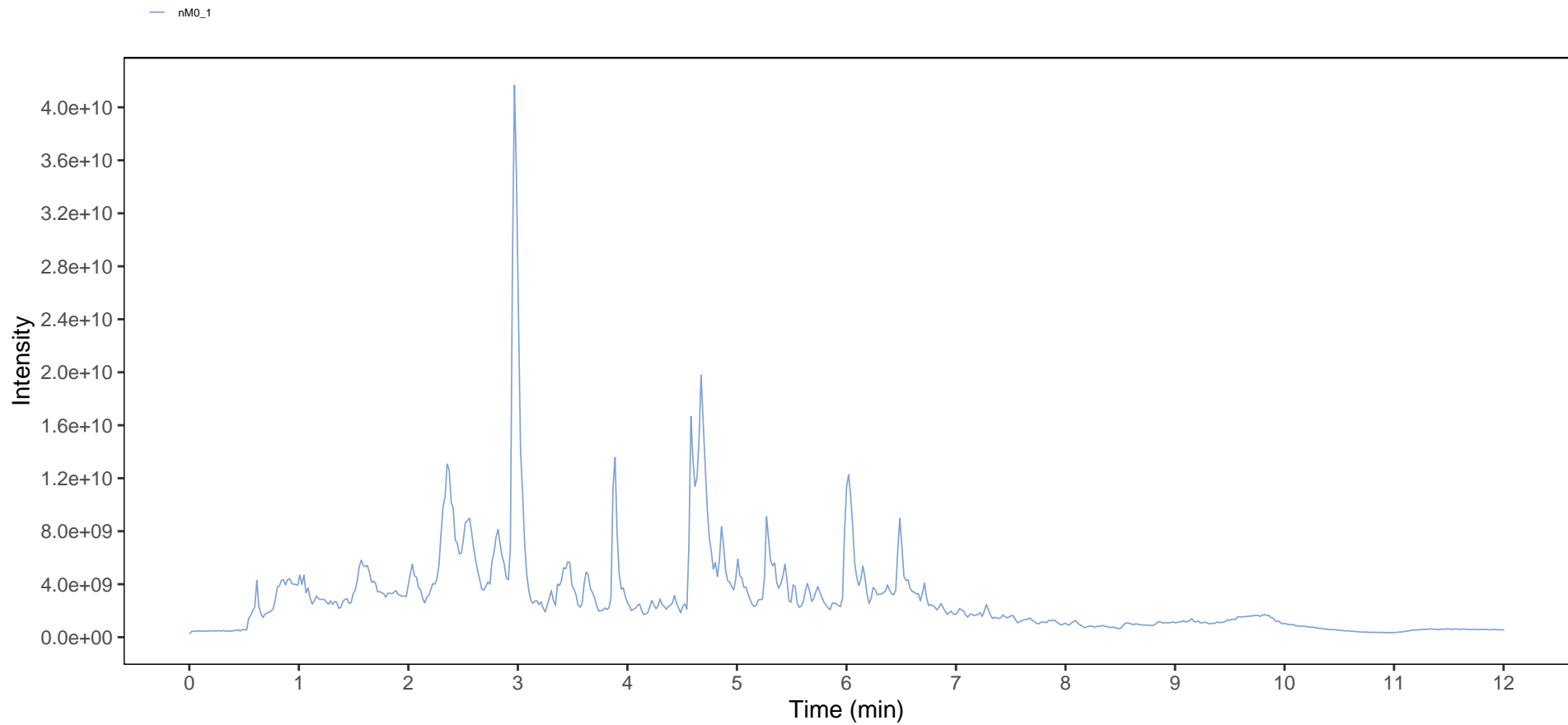

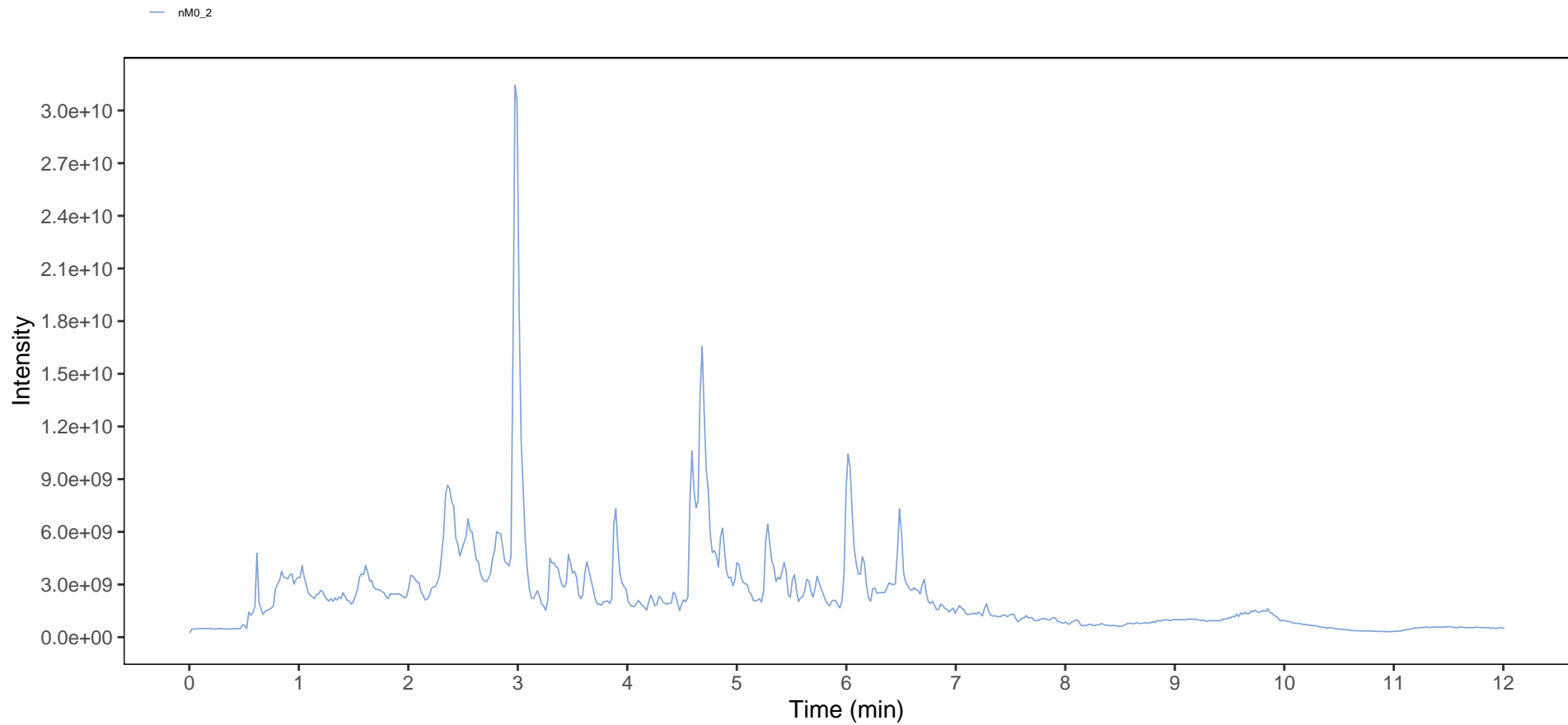

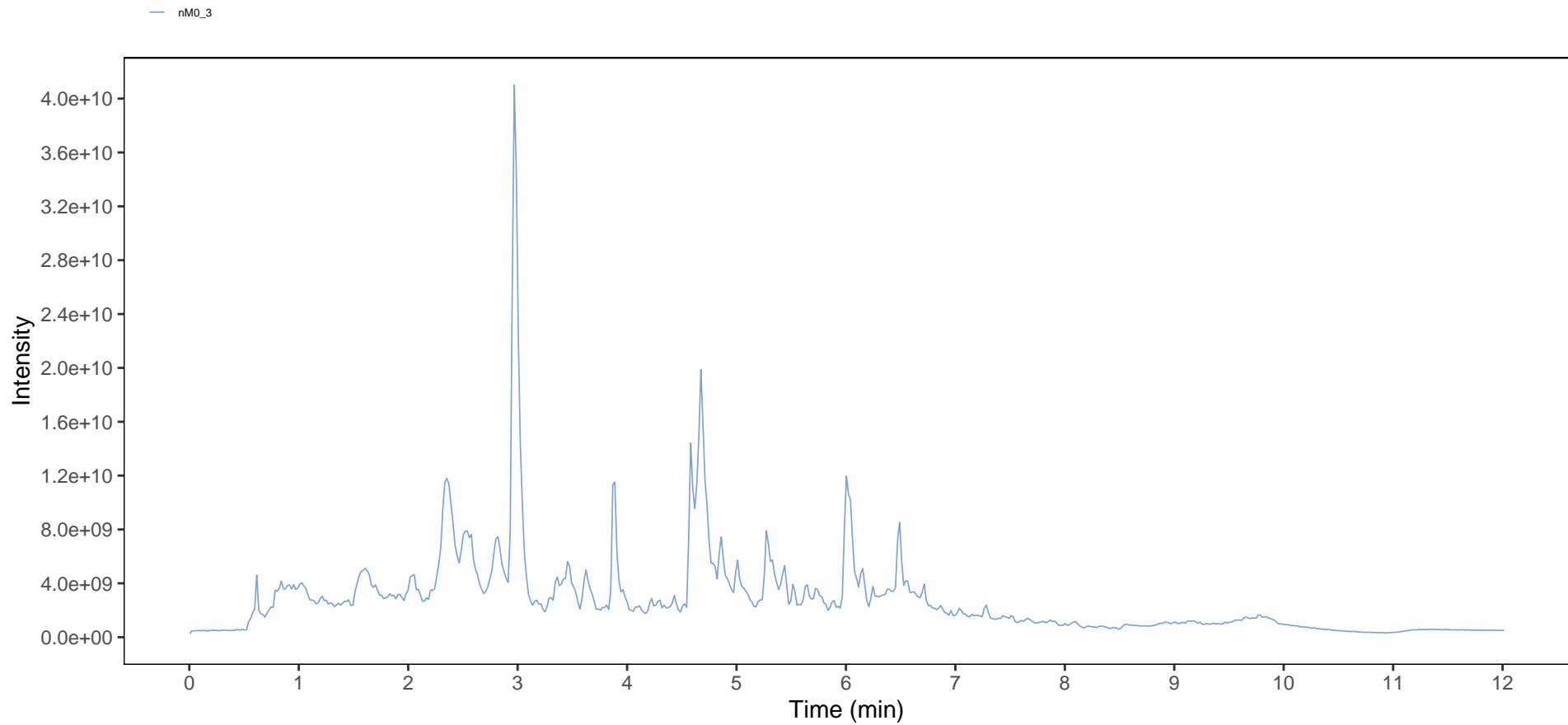

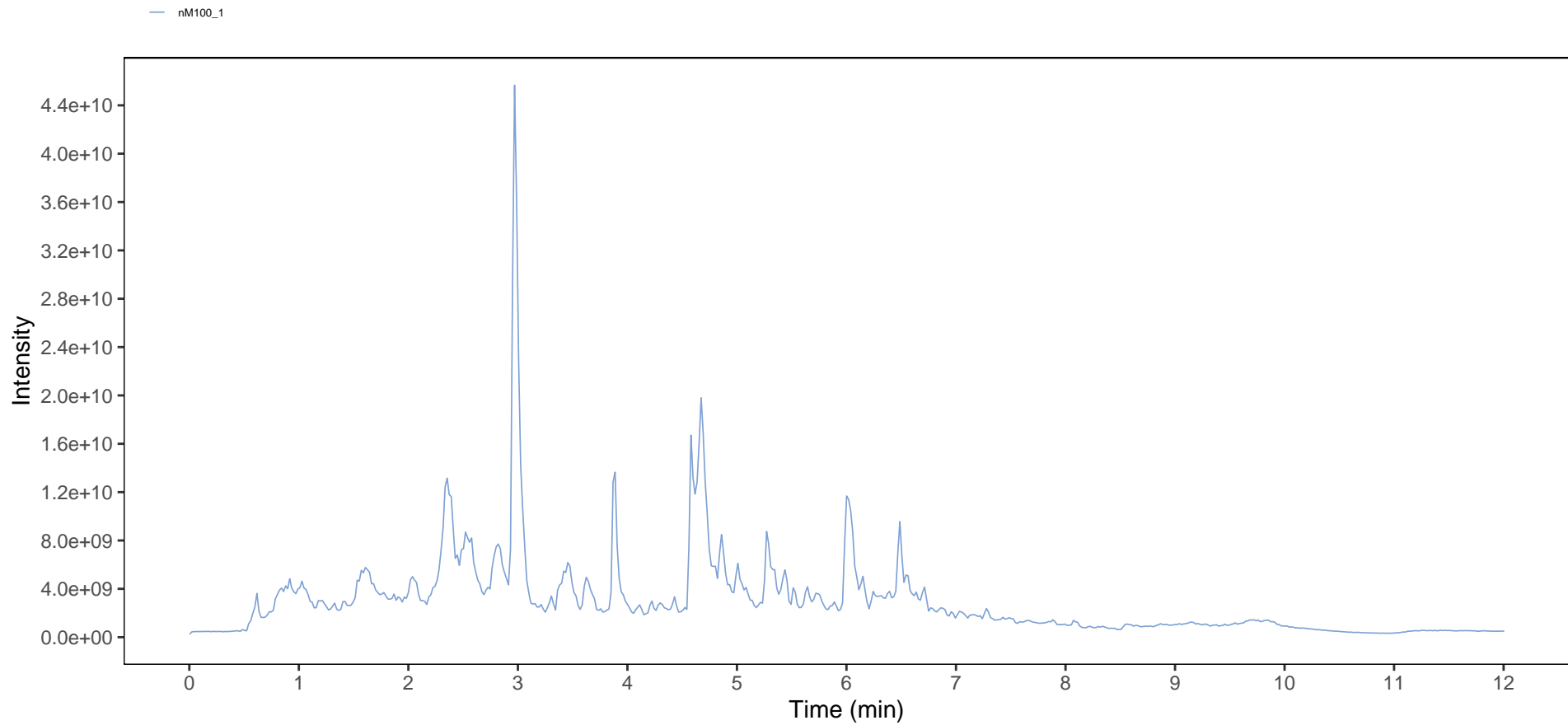

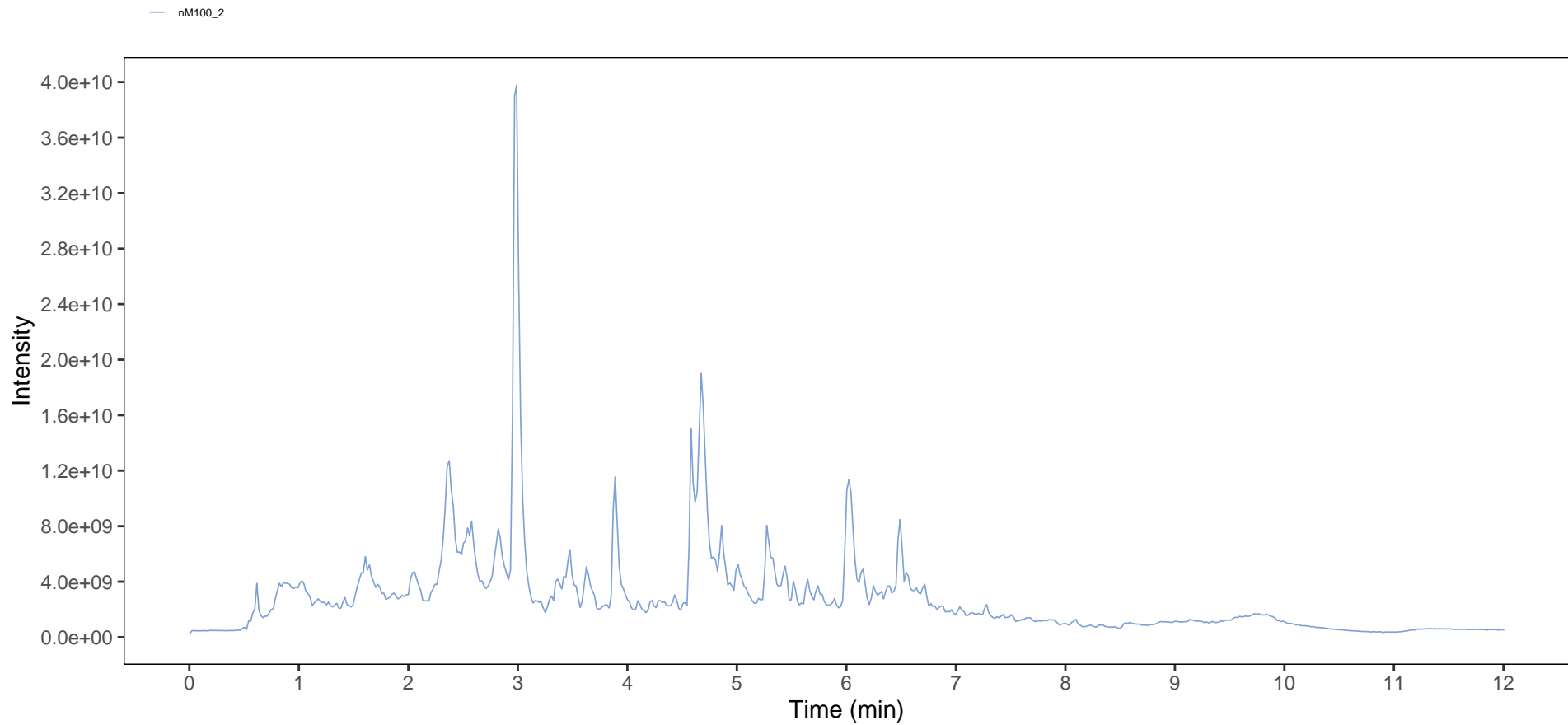

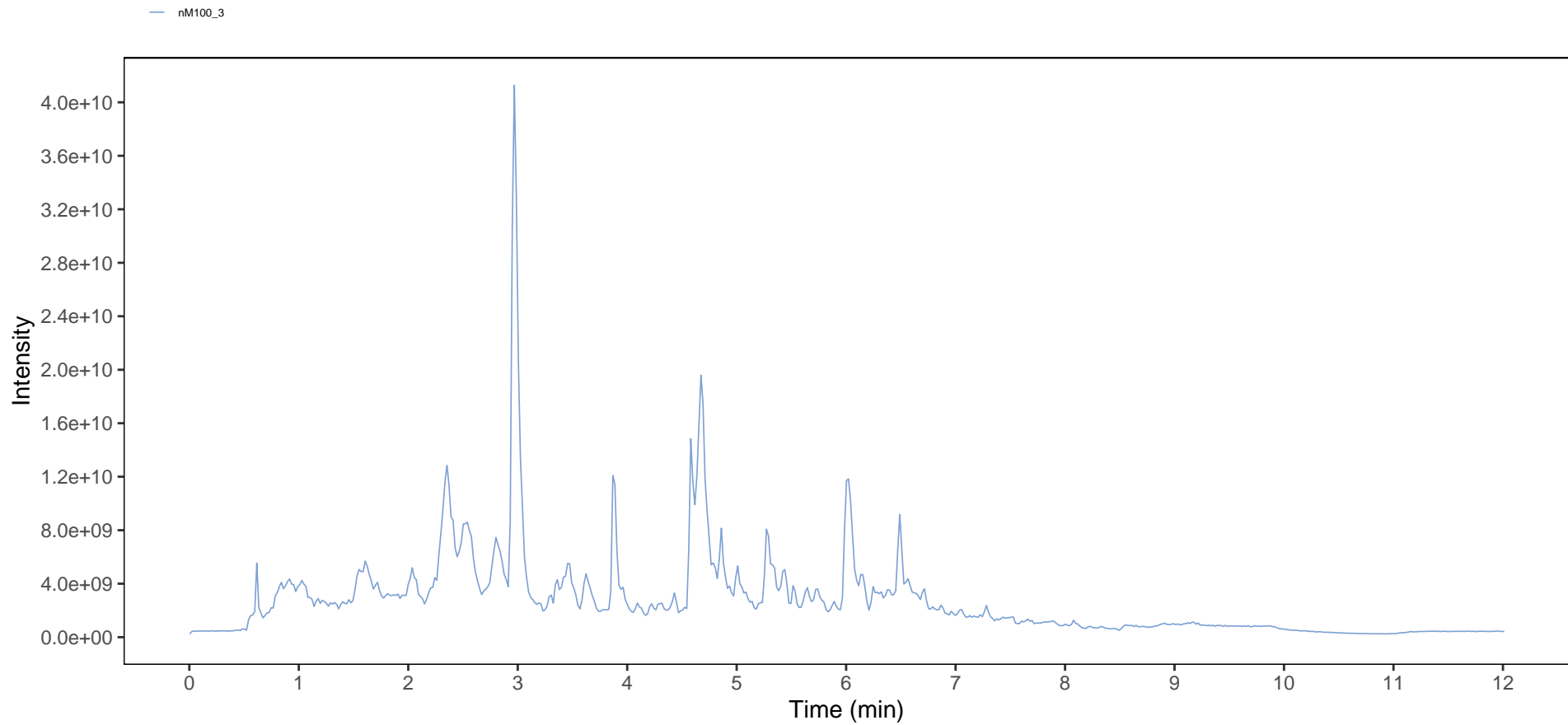

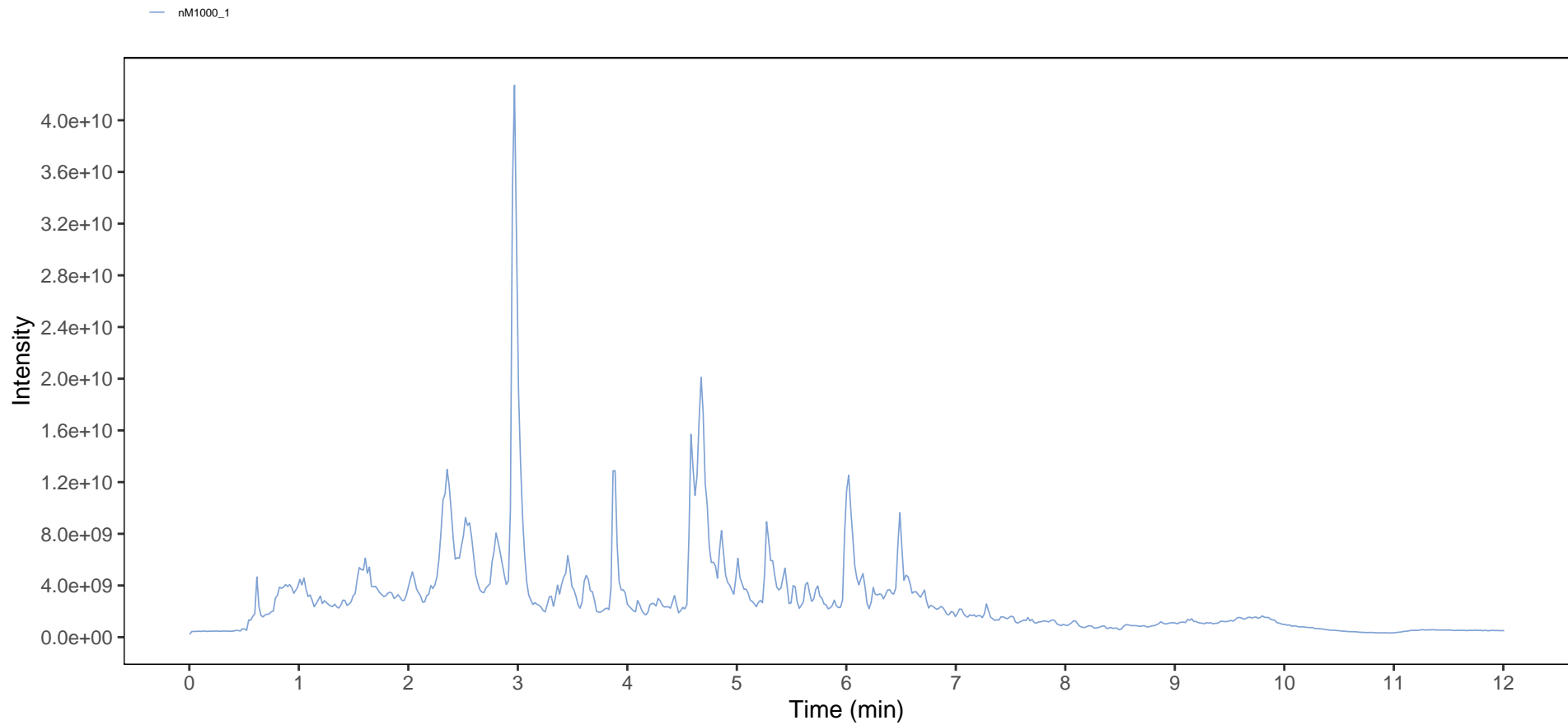

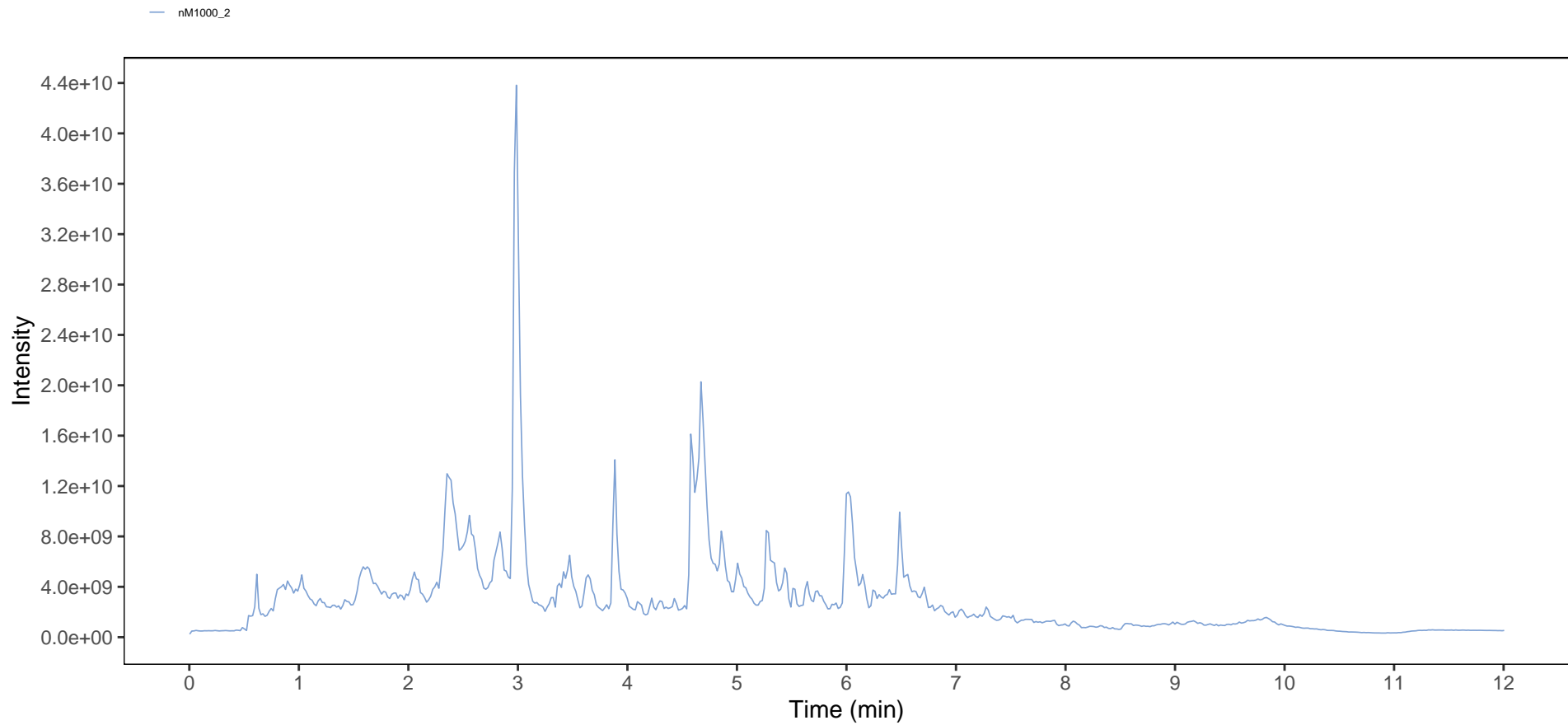

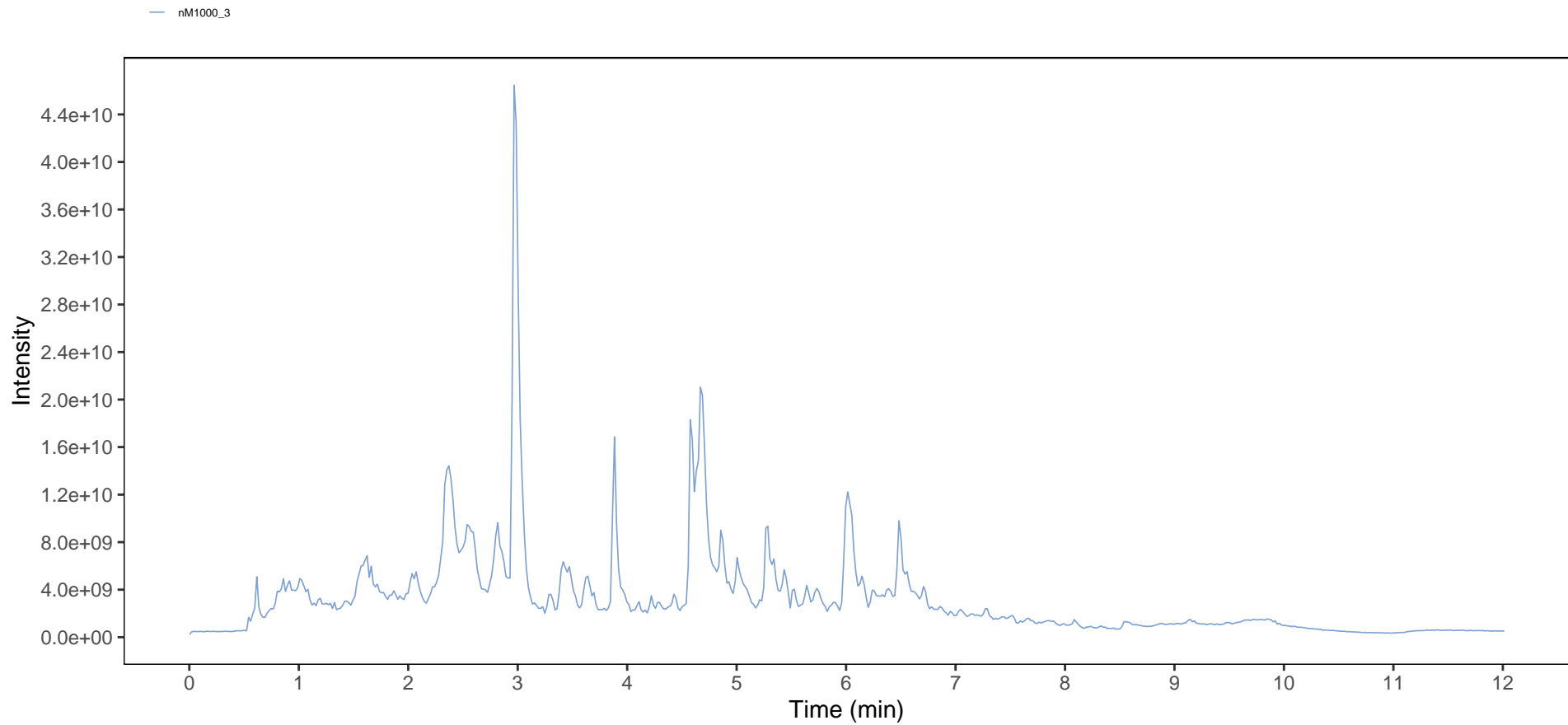

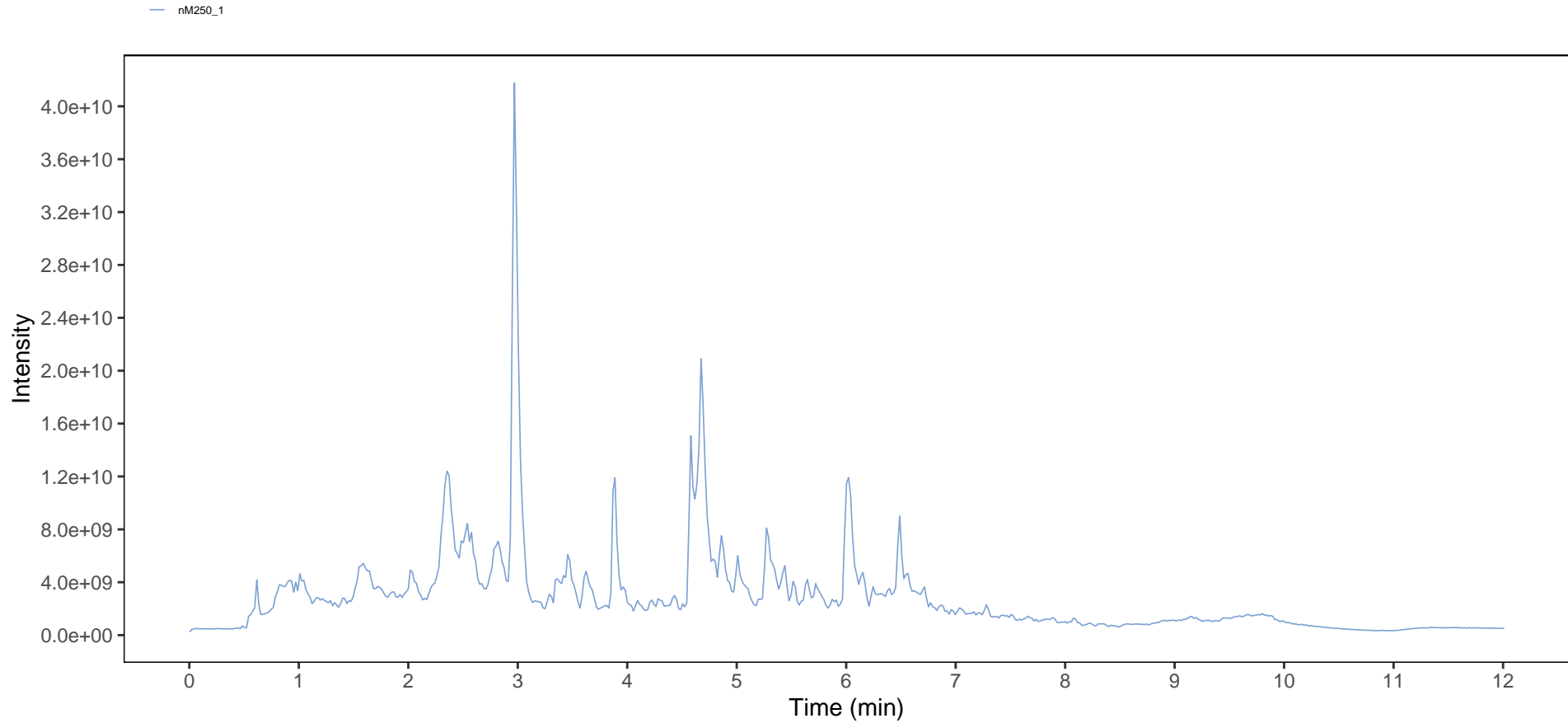

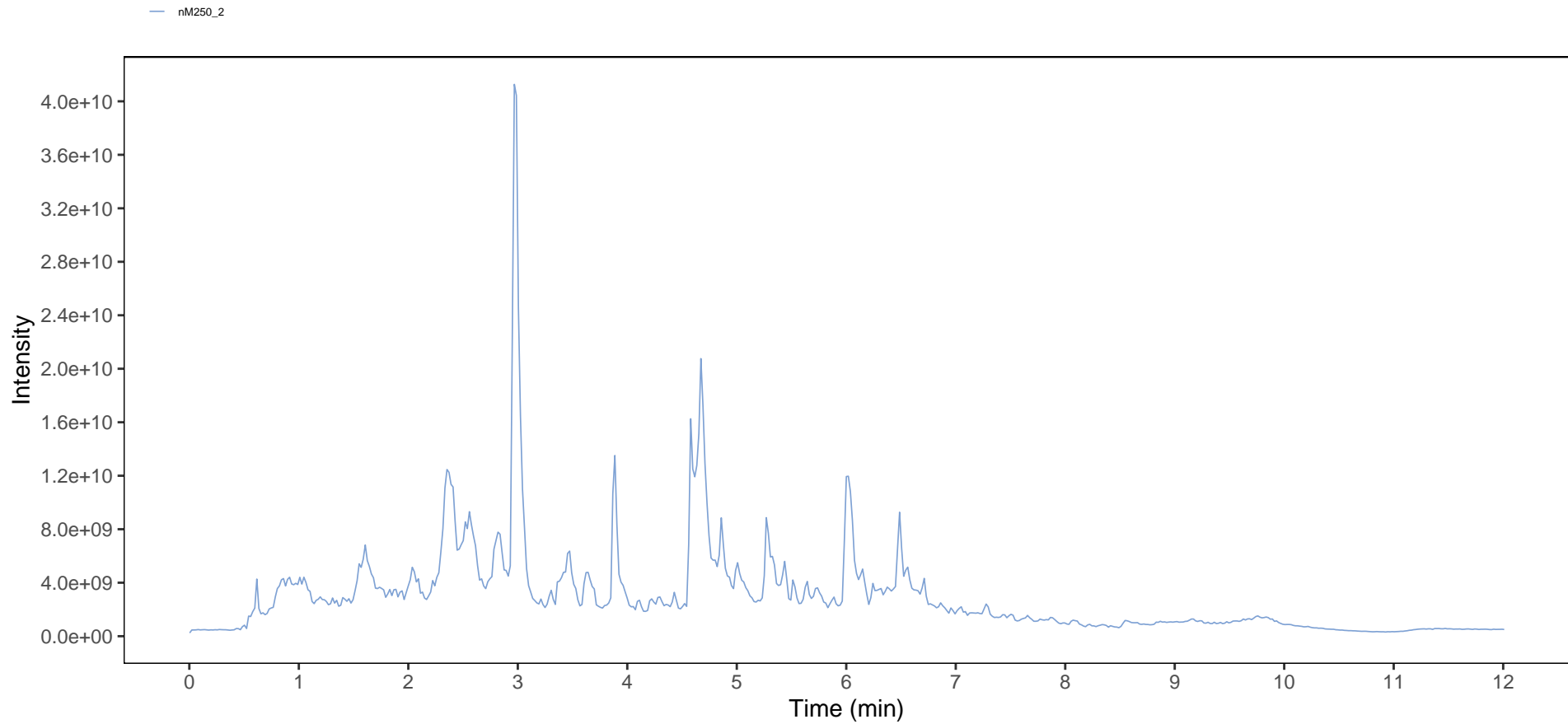

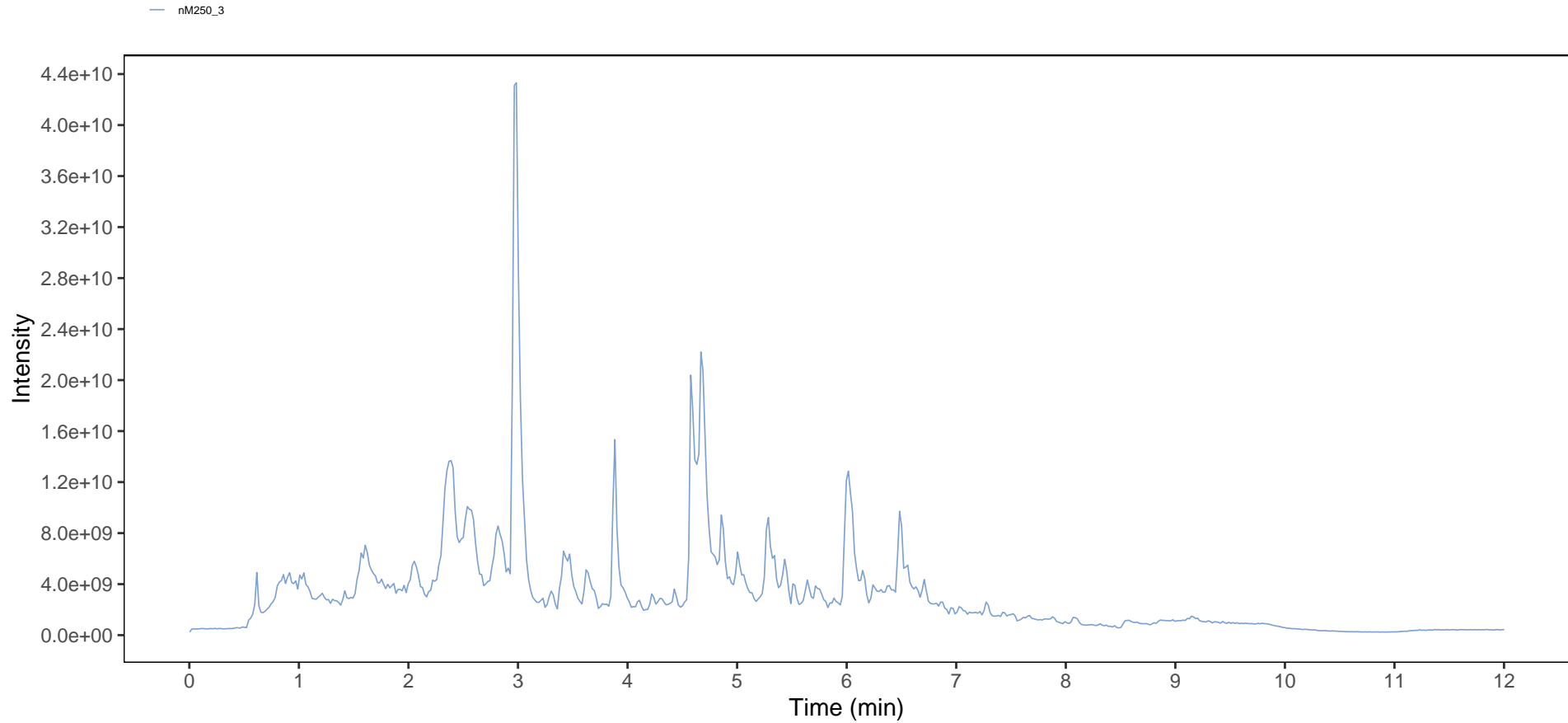

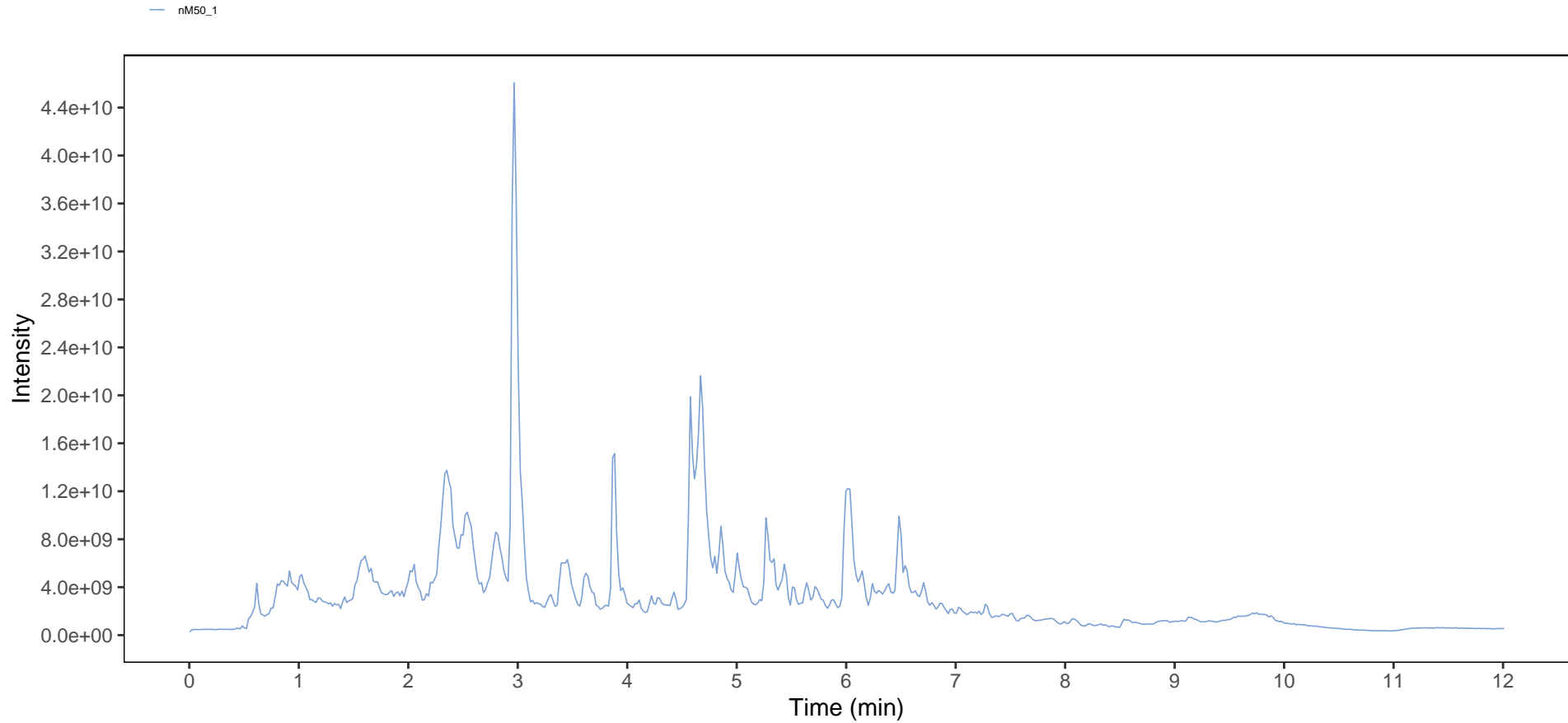

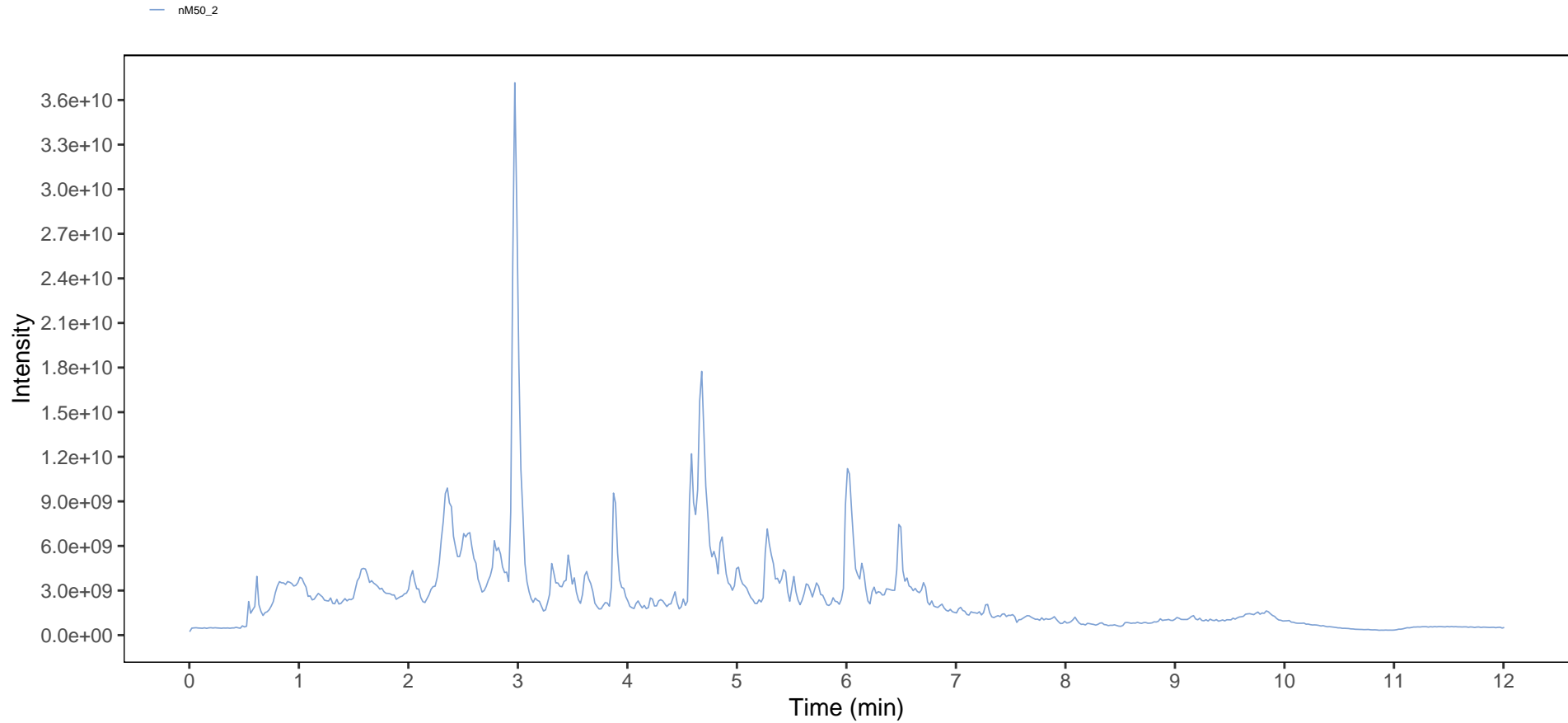

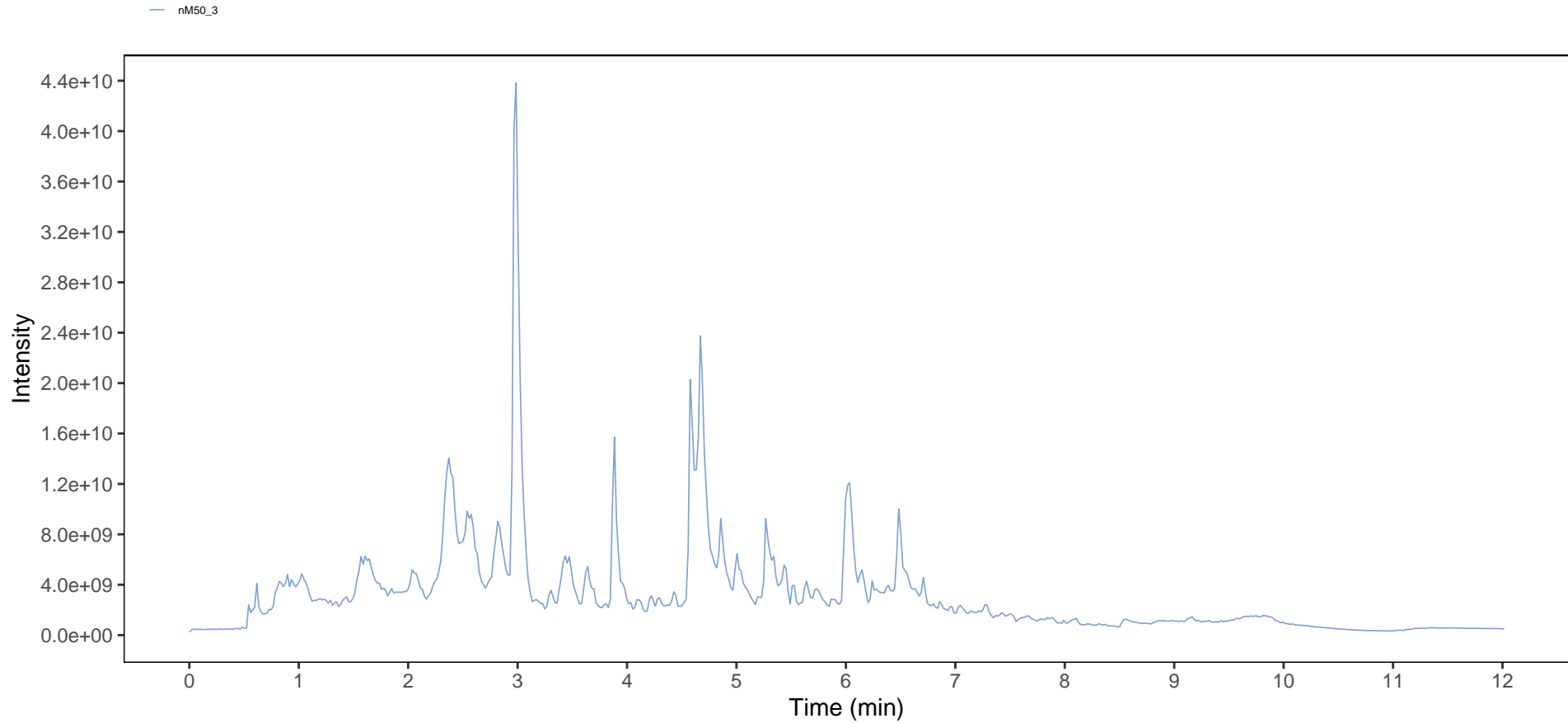

Supplement: Supplementary file 1 [file marinedrugs-20-00556-s001.zip › marinedrugs-1841172-supplementary/POS-T.pdf]
